# Data for the identification of proteins and post-translational modifications of proteins associated to histones H3 and H4 in *S. cerevisiae*, using tandem affinity purification coupled with mass spectrometry

**DOI:** 10.1016/j.dib.2016.01.068

**Published:** 2016-02-05

**Authors:** M Luz Valero, Ramon Sendra, Mercè Pamblanco

**Affiliations:** a Secció de Proteòmica, Servei Central de Suport a la Investigació Experimental (SCSIE), Universitat de València, C/Dr. Moliner 50, 46100 Burjassot, València, Spain; b Departament de Bioquímica i Biologia Molecular, Universitat de València, C/ Dr. Moliner 50, 46100 Burjassot, Valencia, Spain

**Keywords:** Chromatin, Histones, Post-translational modifications, Proteomics, Tandem affinity purification, Yeast

## Abstract

Tandem affinity purification method (TAP) allows the efficient purification of native protein complexes which incorporate a target protein fused with the TAP tag. Purified multiprotein complexes can then be subjected to diverse types of proteomic analyses. Here we describe the data acquired after applying the TAP strategy on histones H3 and H4 coupled with mass spectrometry to identify associated proteins and protein post-translational modifications in the budding yeast, *Saccharomyces cerevisiae*. The mass spectrometry dataset described here consists of 14 files generated from four different analyses in a 5600 Triple TOF (Sciex) by information‐dependent acquisition (IDA) LC–MS/MS. The above files contain information about protein identification, protein relative abundance, and PTMs identification. The instrumental raw data from these files has been also uploaded to the ProteomeXchange Consortium via the PRIDE partner repository, with the dataset identifier PRIDE: PXD002671 and http://dx.doi.org/10.6019/PXD002671. These data are discussed and interpreted in http://dx.doi.org/10.1016/j.jprot.2016.01.004. Valero et al. (2016) [1].

**Specifications Table**

**Table:** 

Subject area	Biology
More specific subject area	Proteomics, protein-protein interactions, post-translational modifications
Type of data	Tables and MS spectra
How data was acquired	LC–MS/MS nanoESI qQTOF Mass spectrometer (5600 Triple TOF, Sciex)
Data format	Analyzed and filtered
Experimental factors	Recombinant yeast cells expressing TAP-tagged histone H3 or H4 were generated. Whole-cell extracts from these cells, grown to OD_600_≅2.0, were prepared and subjected to the tandem affinity purification procedure.
Experimental features	Original tandem affinity purification protocol was applied to whole extracts prepared with two buffers with distinct harsh properties.Affinity purified multiprotein complexes were digested by trypsin and LC–MS/MS analyzed (5600 Triple TOF spectrometer). Proteins and post-translational modifications were identified.
Data source location	Burjassot (València), Spain
Data accessibility	Data are within the article and also on ProteomeXchange Consortium, dataset identifier PRIDE: PXD002671; http://dx.doi.org/10.6019/PXD002671.

**Value of the data**

• The original tandem affinity purification (TAP) method combined with LC–MS/MS analysis can be applied efficiently to histone proteins H3 and H4 in the budding yeast to identify escort proteins and their post-translational modifications.
• Among around 400 proteins associated to H3 and H4, most of them are involved in chromatin dynamics, and H3, H4, H2B and H2A histones, Rtt106p, Spt16p, Pob3p and Psh1p were the most abundant.
• Multiple protein post-translational modifications (PTMs) can be identified on the purified proteins, being some of them new ubiquitination sites.
• Data indicate that serine and threonine residues of yeast histones are also targets of ubiquitination.
• The conditions for MS analyses employed in this work prevent artifacts due to overalkylation by iodoacetamide (IAM).

## 1. Data

1

Proteins co-purifying with yeast TAP-tagged histones H3 and H4 under two different extraction conditions were analyzed by mass spectrometry using LC–MS/MS in IDA mode [1]. The resulting data are presented as lists of proteins and PTMs obtained for each tagged histone and each extraction condition. Spectra of peptides to validate ubiquitination sites are also included. Additionally, results of our test experiments verifying the absence of ubiquitination artifacts by overalkylation are presented as lists of modified peptides.

## 2 Experimental design, materials and methods

### 2.1 Tandem affinity purification (TAP) procedure

The epitope TAP was introduced, at the 3’ end of the ORF by PCR-mediated one-step gene replacement [2] with a tag variant (CBP-T7-TEV-Protein A-KAN MX6) [3], into the genome of BMA64-1A yeast strain cells [4]. Yeast cells, harvested by centrifugation, were cryo-grounded (6 times, 3 min, at maximum speed) by using a MM301 ball mill (Retsch). Whole-cell-extracts were prepared with 2 mL (per g of powered cells) of two extraction buffers which differed in harshness: buffer A (10 mM HEPES, pH 7.9, 210 mM KCl, 1.5 mM MgCl_2_ and 0.5 mM DTT), as previously described [5] and buffer W (40 mM HEPES, pH 7.9, 350 mM NaCl, 10% glycerol and 0.1% Nonidet NP-40) prepared as described [6]. Both extraction buffers contained protease inhibitors (2 mM benzamidine, 1 mM PMSF, 2 mg/mL leupeptine, 2 mg/mL pepstatin A, 2.4 mg/mL chymostatin, and 10 µL/mL Trasylol). The two affinity chromatographies were carried out as described elsewhere [5]. One portion of each solution containing the TAP-purified protein complexes was processed for analysis by LC–MS/MS.

### 2.2 Mass spectrometry analyses

The purified proteins obtained from cells expressing TAP-tagged H3 or H4 and also from non-tagged control cells were processed and identified at the 95% confidence level by LC–MS/MS, as previously described [3]. Eluted proteins were precipitated with 10% trichloroacetic acid (TCA) for 4 h at 5 °C. Precipitated proteins were collected by centrifugation, washed with cold acetone, air dried, and finally dissolved with 20 µL of 100 mM ammonium bicarbonate (ABC). Protein concentration was quantified by using the Qubit protein assay kit (Molecular Probes). Cysteine residues were reduced with 2 mM DL-dithiothreitol (DTT) and the resulting sulfhydryl groups alkylated with 5 mM iodoacetamide (IAM) in 50 mM ammonium bicarbonate (ABC) in the dark at room temperature for a 30-min incubation period. IAM excess was then neutralized by adding DTT at a final concentration of 10 mM and incubated for 30 min at room temperature. Next, proteins were digested with 200 ng of sequencing-grade modified trypsin (Promega) in 50 mM ABC, at 37 °C. The reactions were stopped with trifluoroacetic acid (TFA) at 0.1% and the resulting peptides concentrated in a vacuum concentrator to 0.3 µg/µL. LC–MS/MS analyses were performed with 1.5 μg of each sample.

For LC–MS/MS, the peptide mixtures were loaded onto a trap column (Nano LC Column, 3 μm C18‐CL, 75 μm x 15 cm; Eksigent) and desalted with 0.1% TFA at 3 μL/min for 5 min and then onto an analytical column (LC Column, 3 μm C18‐CL, 75 μm x 25 cm, Eksigent), equilibrated in 5% ACN, 0.1% formic acid (FA). Peptides were eluted with a linear gradient of 5–35% of solvent B in solvent A for 120 min (A: 0.1% FA; B: ACN, 0.1% FA) at a flow rate of 300 nL/min. Purified peptides were analyzed in a nanoESI qQTOF mass spectrometer (5600 TripleTOF, Applied Biosystems Sciex). The Triple TOF was operated in the information‐dependent acquisition (IDA) mode, in which a 0.25‐s TOF MS scan from 350–1250 m/z was performed, followed by 0.05‐s production scans from 100–1500 m/z on the 50 most intense 2–5 charged ions.

### 2.3 Database search and protein identification

Proteins were identified using the Protein-Pilot v4.5 (Sciex) search program. Protein-Pilot default parameters were used to generate peak list directly from 5600 Triple TOF wiff files. The Paragon algorithm of Protein-Pilot was used to search Expasy yeast protein database with the following parameters: trypsin specificity, Cys-carbamidomethylation, and the search effort set to thorough.

Supplementary Table S1 is a descriptive list of all supplied excel files (Tables S2–S14).

Supplementary Tables S2–S6 show the results of the bioinformatics analysis of the identified protein mixtures. These tables contain the total proteins co-purifying with tagged H3 or H4 and identified with FDR below 1% and a confidence level above 95%. For modification analysis all peptide matches were initially filtered based on Protein-Pilot peptide confidence (≥95%) followed by manual validation of all spectra with the aid of Protein-Pilot following the criteria described below.

Mascot v2.4 (Matrix Science) searches were run to obtain emPAI values, in order to quantify approximately the relative content of the co-purified proteins (Supplementary Tables S7–S10). The peak lists were generated directly from wiff files by Protein-Pilot. Database search was done in Expasy *Saccharomyces cerevisiae* (7641 sequences) protein. The search parameters were set to tryptic specificity, Cys-carbamidomethylation, one missed cleavage and a tolerance in the mass measurement of 50 ppm in MS mode and 0.5 Da for MS/MS ions. Error tolerant option was set for searching variable modifications. The significant threshold was set to *p*<0.05.

The MS raw data (wiff files, peak lists, and protein identification results) have been deposited in the ProteomeXchange Consortium [7] via the PRIDE partner repository with the dataset identifier PRIDE: PXD002671; http://dx.doi.org/10.6019/PXD002671.

### 2.4 Manual validation of ubiquitination

Manual validation of ubiquitination was performed for the most representative proteins in our analyses: histone H3, histone H4, histone H2B, histone H2A, Rtt106p, Pob3p, Spt16p and Psh1p. For this, the MS/MS spectra of the assigned modified peptides were extracted by Protein-Pilot, and each ubiquitin modification was only accepted if two of the following three criteria were met: 1) Protein-Pilot identified the modified peptide with a 99% confidence level; 2) the complete b/y ions series was found in the MS/MS spectrum; and 3) the immonium ion of the ubiquitinated residue was found in the MS/MS spectrum. Analysis and verification of ubiquitinated peptides can be found in the Supplementary File “Spectrum ubiquitination validation” and Supplementary Table S11.

### 2.5 Side reaction control experiments

Other authors have reported that IAM may modify amino acid residues other than Cys [8], and induce artefactual adducts that mimic ubiquitination modification in the MS analysis [9]. To test whether under our experimental conditions IAM generates undesired side products, we carried out control experiments with histones purified by acid extraction from isolated chromatin of chicken erythrocytes and yeast cells [10]. Purified histones were processed as described above using 5 mM IAM and 10 mM DTT as neutralizing agent or by following a standard procedure with 55 mM IAM and without neutralizing reagent [9]. As an additional control, 55 mM IAM was substituted by chloroacetamide (ClAM), an alternative alkylating reagent which does not produce ubiquitination artifacts [9]. The identified modified peptides resulting from the artifacts detection experiments with purified histones are listed in Supplementary Tables S12–S14.

## Footnotes

Appendix A Supplementary data associated with this article can be found in the online version at doi:10.1016/j.dib.2016.01.068.
