# Supplementary material for: Data for the identification of proteins and post-translational modifications of proteins associated to histones H3 and H4 in S. cerevisiae, using tandem affinity purification coupled with mass spectrometry
Source: Data Brief. 2016 Feb 5;6:965–9. doi: 10.1016/j.dib.2016.01.068 (PMC4758224; doi:10.1016/j.dib.2016.01.068)
Supplement: Supplementary file 1 — Supplementary material [file mmc1.doc]

**Conflict of interest**

All the authors declare no conflict of interest.
