# Supplementary material for: Data for the identification of proteins and post-translational modifications of proteins associated to histones H3 and H4 in S. cerevisiae, using tandem affinity purification coupled with mass spectrometry
Source: Data Brief. 2016 Feb 5;6:965–9. doi: 10.1016/j.dib.2016.01.068 (PMC4758224; doi:10.1016/j.dib.2016.01.068)

INFORMATION :
Labelled MSMS spectrum of the ubiquitinated peptide
Zoom of the MSMS spectrum showing the immonium ion of Kub/Sub or Tub marked with grey line.
Table with the theoretical values of y/b and immonium ions for ubiquitinated peptide, in green matched residues are shown.

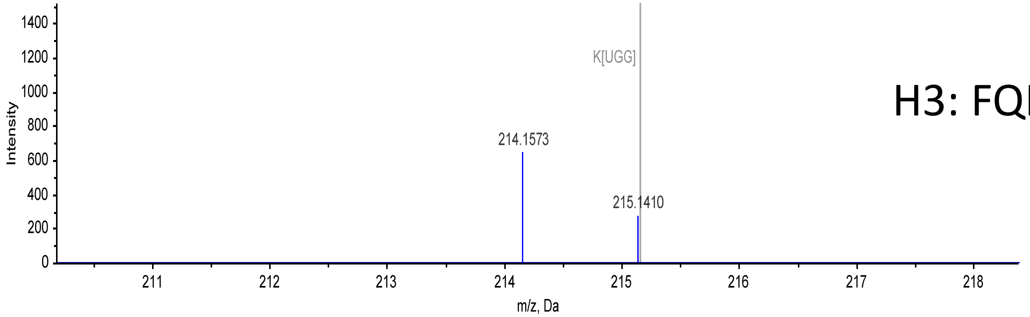

H3: FQKubSTELLIR

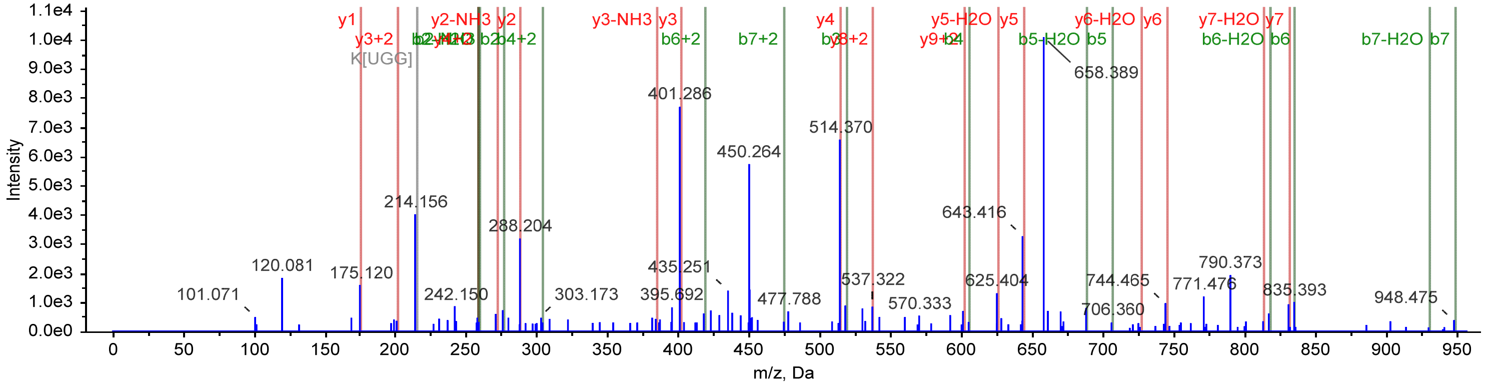

Residue	Immonium	b	b+2	b-H2O	b-NH3	y	y+2	y-H2O	y-NH3
F	120.08	148.08	74.54	130.07	131.05	1348.76	674.88	1330.75	1331.73
Q	101.07	276.13	138.57	258.12	259.11	1201.69	601.35	1183.68	1184.66
K[UGG]	215.15	518.27	259.64	500.26	501.25	1073.63	537.32	1055.62	1056.60
S	60.04	605.30	303.16	587.29	588.28	831.49	416.25	813.48	814.47
T	74.06	706.35	353.68	688.34	689.33	744.46	372.73	726.45	727.43
E	102.06	835.39	418.20	817.38	818.37	643.41	322.21	625.40	626.39
L	86.10	948.48	474.74	930.47	931.45	514.37	257.69	496.36	497.34
L	86.10	1061.56	531.28	1043.55	1044.54	401.29	201.15	383.28	384.26
I	86.10	1174.65	587.83	1156.64	1157.62	288.20	144.61	270.19	271.18
R	129.11	1330.75	665.88	1312.74	1313.72	175.12	88.06	157.11	158.09

H3: FQSSAIGALQESVEAYLVSLFEDTNLAAIHAKubR

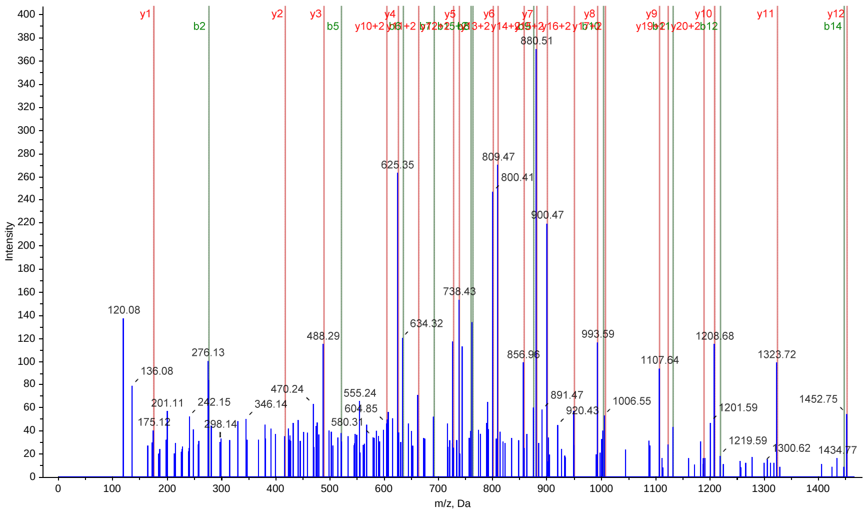

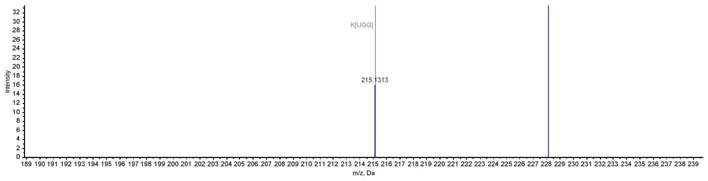

Residue	Immonium	b	b+2	b-H2O	b-NH3	y	y+2	y-H2O	y-NH3
F	120.0808	148.0757	74.5415	130.0651	131.0491	3692.892	1846.95	3674.881	3675.865
Q	101.0709	276.1343	138.5708	258.1237	259.1077	3545.824	1773.415	3527.813	3528.797
S	60.0444	363.1663	182.0868	345.1557	346.1397	3417.765	1709.386	3399.754	3400.738
S	60.0444	450.1983	225.6028	432.1878	433.1718	3330.733	1665.87	3312.722	3313.706
A	44.0495	521.2354	261.1214	503.2249	504.2089	3243.701	1622.354	3225.69	3226.674
I	86.0964	634.3195	317.6634	616.3089	617.293	3172.664	1586.836	3154.653	3155.637
G	30.0338	691.341	346.1741	673.3304	674.3144	3059.58	1530.294	3041.569	3042.553
A	44.0495	762.3781	381.6927	744.3675	745.3515	3002.558	1501.783	2984.548	2985.532
L	86.0964	875.4621	438.2347	857.4516	858.4356	2931.521	1466.264	2913.511	2914.495
Q	101.0709	1003.521	502.264	985.5102	986.4942	2818.437	1409.722	2800.426	2801.41
E	102.055	1132.563	566.7853	1114.553	1115.5368	2690.378	1345.693	2672.368	2673.352
S	60.0444	1219.595	610.3013	1201.585	1202.5688	2561.336	1281.172	2543.325	2544.309
V	72.0808	1318.664	659.8355	1300.653	1301.6372	2474.304	1237.656	2456.293	2457.277
E	102.055	1447.706	724.3568	1429.696	1430.6798	2375.235	1188.121	2357.225	2358.209
A	44.0495	1518.744	759.8754	1500.733	1501.7169	2246.193	1123.6	2228.182	2229.166
Y	136.0757	1681.807	841.407	1663.796	1664.7802	2175.156	1088.082	2157.145	2158.129
L	86.0964	1794.891	897.9491	1776.88	1777.8643	2012.092	1006.55	1994.082	1995.066
V	72.0808	1893.959	947.4833	1875.949	1876.9327	1899.008	950.0078	1880.998	1881.982
S	60.0444	1980.991	990.9993	1962.981	1963.9647	1799.94	900.4736	1781.929	1782.913
L	86.0964	2094.075	1047.541	2076.065	2077.0488	1712.908	856.9576	1694.897	1695.881
F	120.0808	2241.144	1121.076	2223.133	2224.1172	1599.824	800.4155	1581.813	1582.797
E	102.055	2370.186	1185.597	2352.176	2353.1598	1452.755	726.8813	1434.745	1435.729
D	88.0393	2485.213	1243.11	2467.203	2468.1868	1323.713	662.36	1305.702	1306.686
T	74.06	2586.261	1293.634	2568.25	2569.2344	1208.686	604.8466	1190.675	1191.659
N	87.0553	2700.304	1350.656	2682.293	2683.2774	1107.638	554.3227	1089.628	1090.612
L	86.0964	2813.388	1407.198	2795.377	2796.3614	993.5952	497.3013	975.5847	976.5687
A	44.0495	2884.425	1442.716	2866.415	2867.3985	880.5112	440.7592	862.5006	863.4846
A	44.0495	2955.462	1478.235	2937.452	2938.4357	809.474	405.2407	791.4635	792.4475
I	86.0964	3068.546	1534.777	3050.536	3051.5197	738.4369	369.7221	720.4264	721.4104
H	110.0713	3205.605	1603.306	3187.595	3188.5786	625.3529	313.1801	607.3423	608.3263
A	44.0495	3276.642	1638.825	3258.632	3259.6158	488.294	244.6506	470.2834	471.2674
K[UGG]	215.1503	3518.78	1759.894	3500.77	3501.7536	417.2568	209.1321	399.2463	400.2303
R	129.1135	3674.881	1837.944	3656.871	3657.8548	175.119	88.0631	157.1084	158.0924

H3: YKubPGTVALR

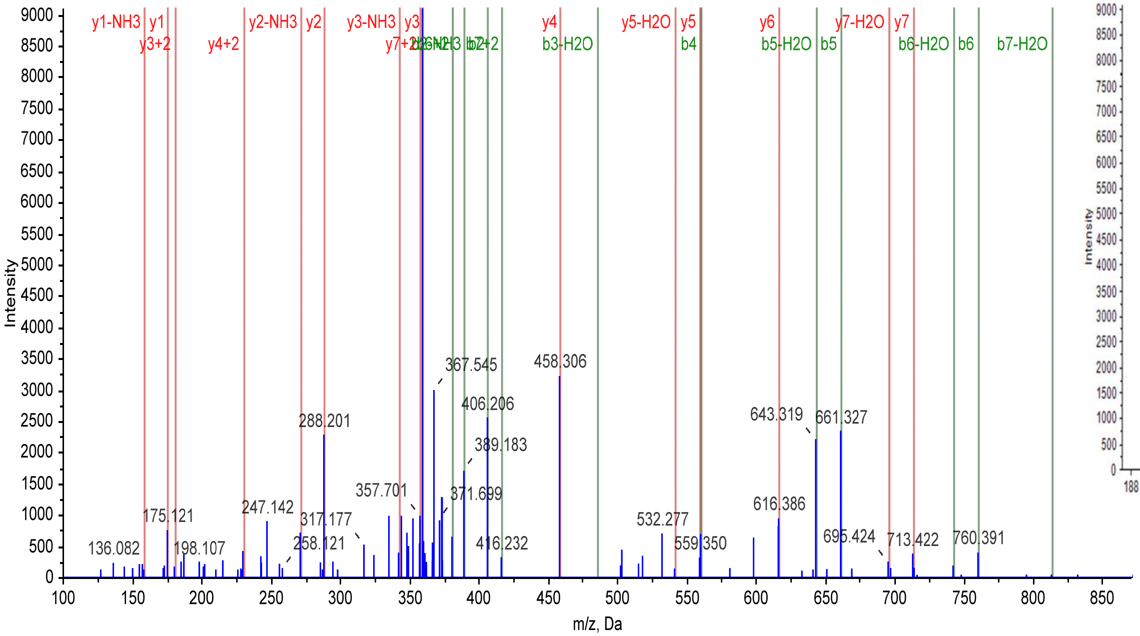

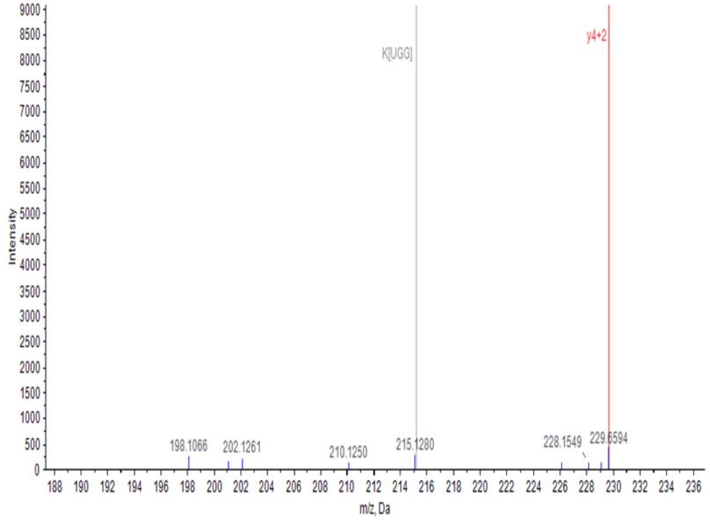

Residue	Immonium	b	b+2	b-H2O	b-NH3	y	y+2	y-H2O	y-NH3
Y	136.0757	164.0706	82.5389	146.06	147.0441	1118.632	559.8195	1100.621	1101.605
K[UGG]	215.1503	406.2085	203.6079	388.1979	389.1819	955.5683	478.2878	937.5578	938.5418
P	70.0651	503.2613	252.1343	485.2507	486.2347	713.4304	357.2189	695.4199	696.4039
G	30.0338	560.2827	280.645	542.2722	543.2562	616.3777	308.6925	598.3671	599.3511
T	74.06	661.3304	331.1688	643.3198	644.3039	559.3562	280.1817	541.3457	542.3297
V	72.0808	760.3988	380.703	742.3883	743.3723	458.3085	229.6579	440.298	441.282
A	44.0495	831.4359	416.2216	813.4254	814.4094	359.2401	180.1237	341.2296	342.2136
L	86.0964	944.52	472.7636	926.5094	927.4934	288.203	144.6051	270.1925	271.1765
R	129.1135	1100.621	550.8142	1082.611	1083.595	175.119	88.0631	157.1084	158.0924

H4: AVLKubSFLESVIR

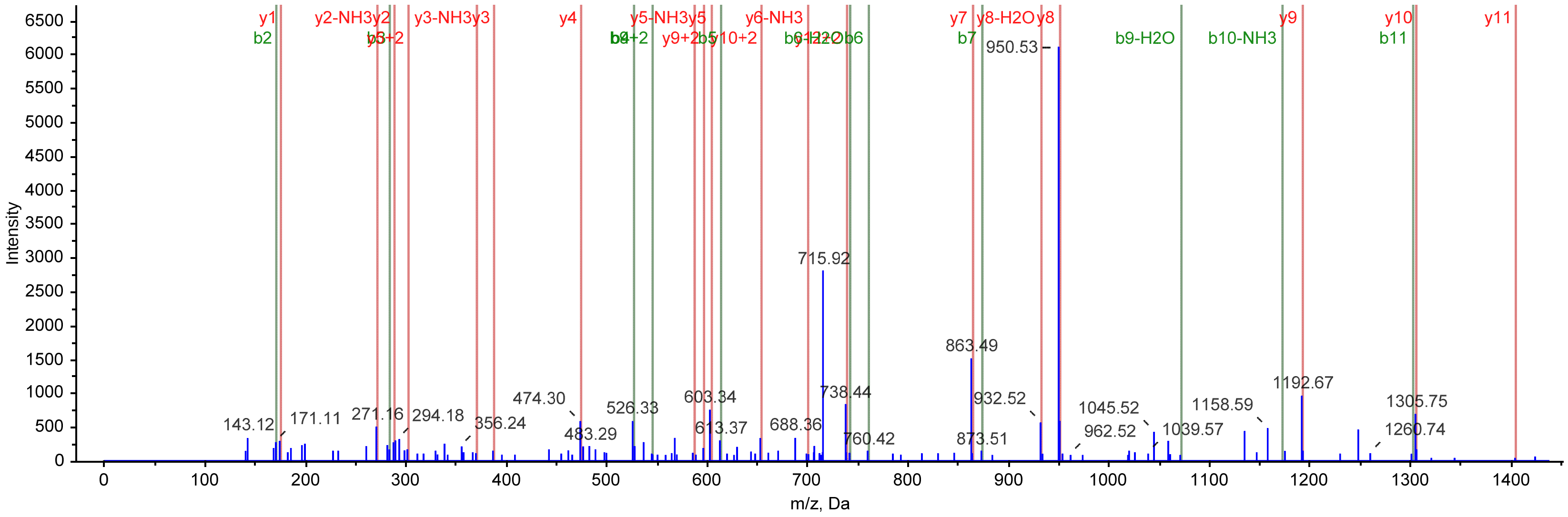

Residue	Immonium	b	b+2	b-H2O	b-NH3	y	y+2	y-H2O	y-NH3
A	44.0495	72.0444	36.5258	54.0338	55.0178	1475.858	738.4327	1457.848	1458.832
V	72.0808	171.1128	86.06	153.1022	154.0863	1404.821	702.9141	1386.81	1387.794
L	86.0964	284.1969	142.6021	266.1863	267.1703	1305.753	653.3799	1287.742	1288.726
K[UGG]	215.1503	526.3348	263.671	508.3242	509.3082	1192.668	596.8379	1174.658	1175.642
S	60.0444	613.3668	307.187	595.3562	596.3402	950.5306	475.7689	932.52	933.504
F	120.0808	760.4352	380.7212	742.4246	743.4087	863.4985	432.2529	845.488	846.472
L	86.0964	873.5193	437.2633	855.5087	856.4927	716.4301	358.7187	698.4196	699.4036
E	102.055	1002.562	501.7846	984.5513	985.5353	603.3461	302.1767	585.3355	586.3195
S	60.0444	1089.594	545.3006	1071.583	1072.567	474.3035	237.6554	456.2929	457.2769
V	72.0808	1188.662	594.8348	1170.652	1171.636	387.2714	194.1394	369.2609	370.2449
I	86.0964	1301.746	651.3768	1283.736	1284.72	288.203	144.6051	270.1925	271.1765
R	129.1135	1457.848	729.4274	1439.837	1440.821	175.119	88.0631	157.1084	158.0924

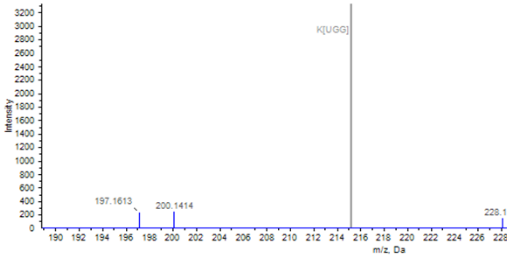

H4: DNIQGITKubPAIR

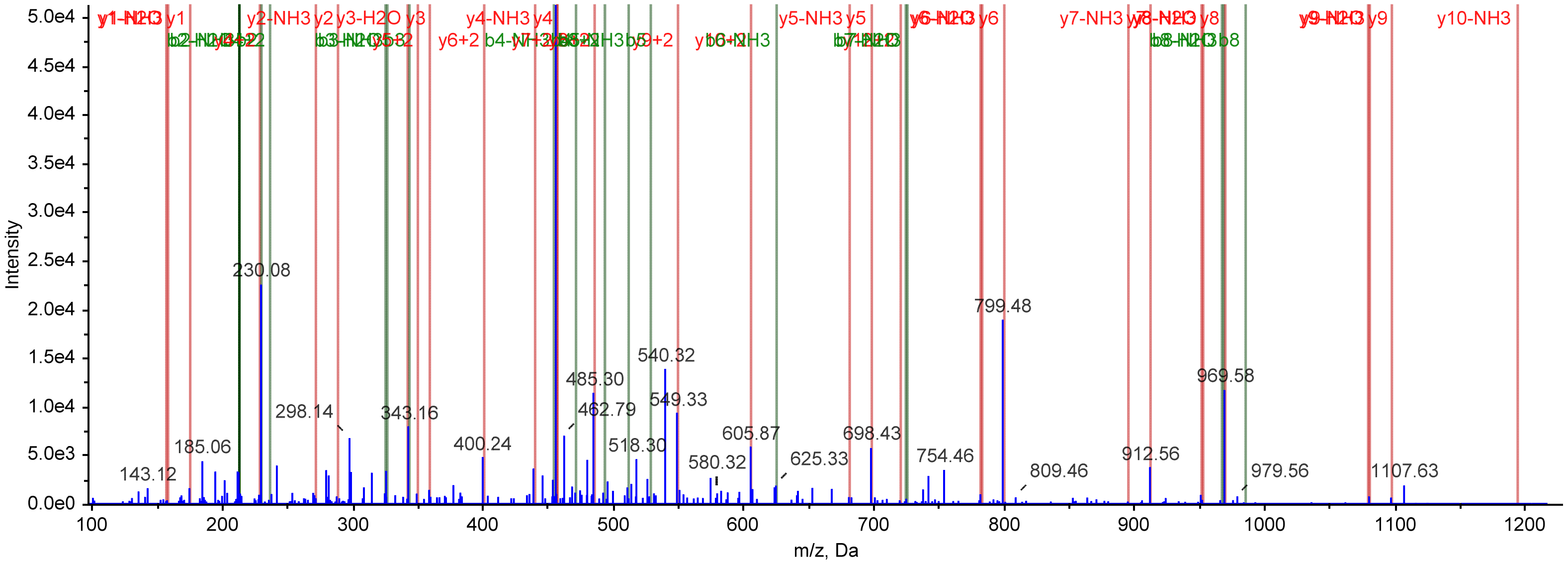

Residue	Immonium	b	b+2	b-H2O	b-NH3	y	y+2	y-H2O	y-NH3
D	88.0393	116.0342	58.5207	98.0237	99.0077	1439.797	720.4019	1421.786	1422.77
N	87.0553	230.0771	115.5422	212.0666	213.0506	1324.77	662.8884	1306.759	1307.743
I	86.0964	343.1612	172.0842	325.1506	326.1347	1210.727	605.867	1192.716	1193.7
Q	101.0709	471.2198	236.1135	453.2092	454.1932	1097.643	549.3249	1079.632	1080.616
G	30.0338	528.2413	264.6243	510.2307	511.2147	969.584	485.2956	951.5734	952.5574
I	86.0964	641.3253	321.1663	623.3148	624.2988	912.5625	456.7849	894.552	895.536
T	74.06	742.373	371.6901	724.3624	725.3464	799.4785	400.2429	781.4679	782.4519
K[UGG]	215.1503	984.5109	492.7591	966.5003	967.4843	698.4308	349.719	680.4202	681.4042
P	70.0651	1081.564	541.2855	1063.553	1064.537	456.2929	228.6501	438.2823	439.2663
A	44.0495	1152.601	576.804	1134.59	1135.574	359.2401	180.1237	341.2296	342.2136
I	86.0964	1265.685	633.3461	1247.674	1248.658	288.203	144.6051	270.1925	271.1765
R	129.1135	1421.786	711.3966	1403.775	1404.759	175.119	88.0631	157.1084	158.0924

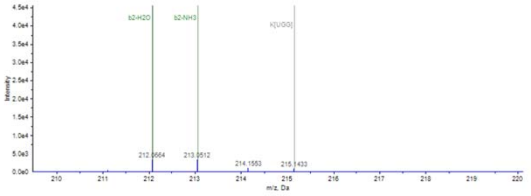

H4: DSVTYTEHAKubR

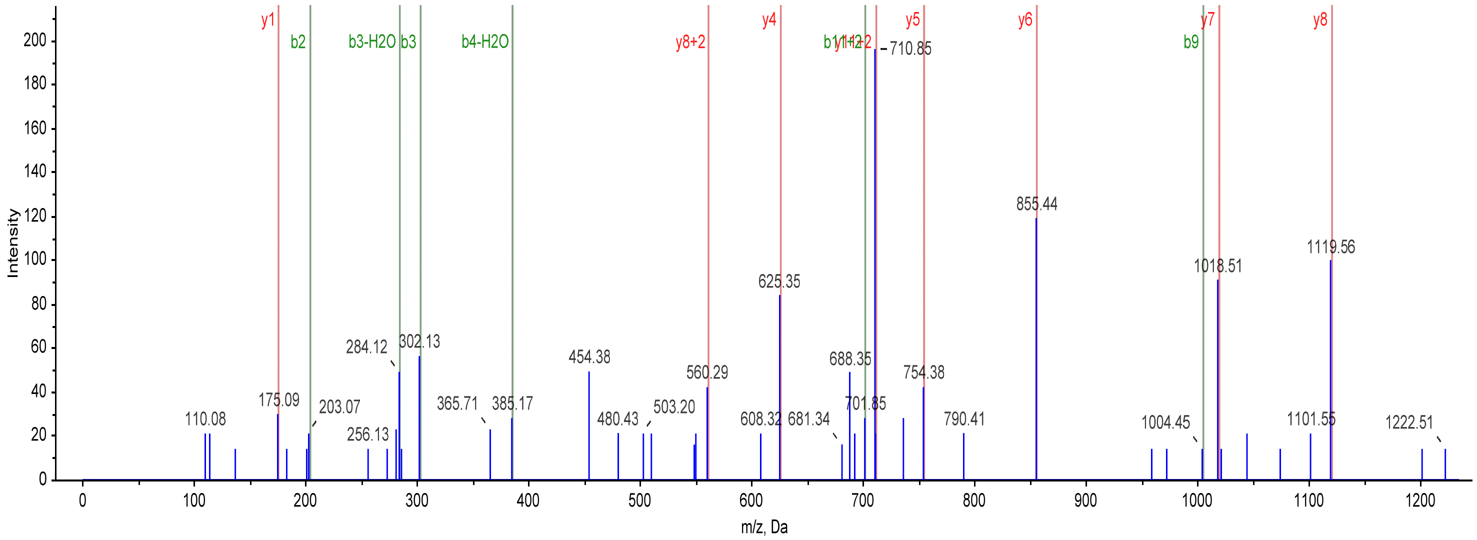

Residue	Immonium	b	b+2	b-H2O	y	y+2	y-H2O	y-NH3
D	88.0393	116.0342	58.5207	98.0237	1420.682	710.8444	1402.671	1403.655
S	60.0444	203.0662	102.0368	185.0557	1305.655	653.3309	1287.644	1288.628
V	72.0808	302.1347	151.571	284.1241	1218.623	609.8149	1200.612	1201.596
T	74.06	403.1823	202.0948	385.1718	1119.554	560.2807	1101.544	1102.528
Y	136.0757	566.2457	283.6265	548.2351	1018.507	509.7569	1000.496	1001.48
T	74.06	667.2933	334.1503	649.2828	855.4431	428.2252	837.4326	838.4166
E	102.055	796.3359	398.6716	778.3254	754.3955	377.7014	736.3849	737.3689
H	110.0713	933.3949	467.2011	915.3843	625.3529	313.1801	607.3423	608.3263
A	44.0495	1004.432	502.7196	986.4214	488.294	244.6506	470.2834	471.2674
K[UGG]	215.1503	1246.57	623.7886	1228.559	417.2568	209.1321	399.2463	400.2303
R	129.1135	1402.671	701.8391	1384.66	175.119	88.0631	157.1084	158.0924

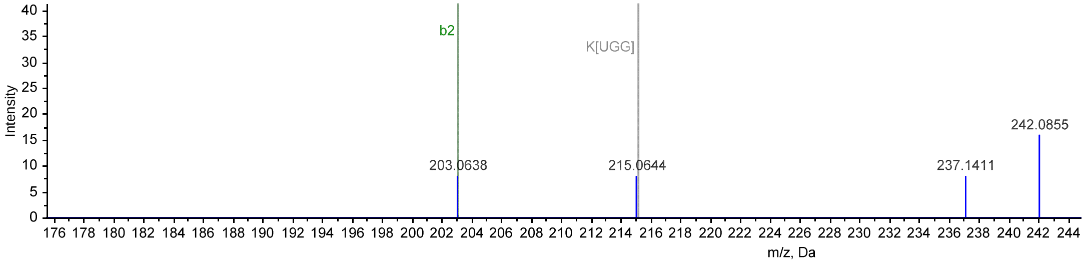

H4:KubILRDNIQGITKPAIR

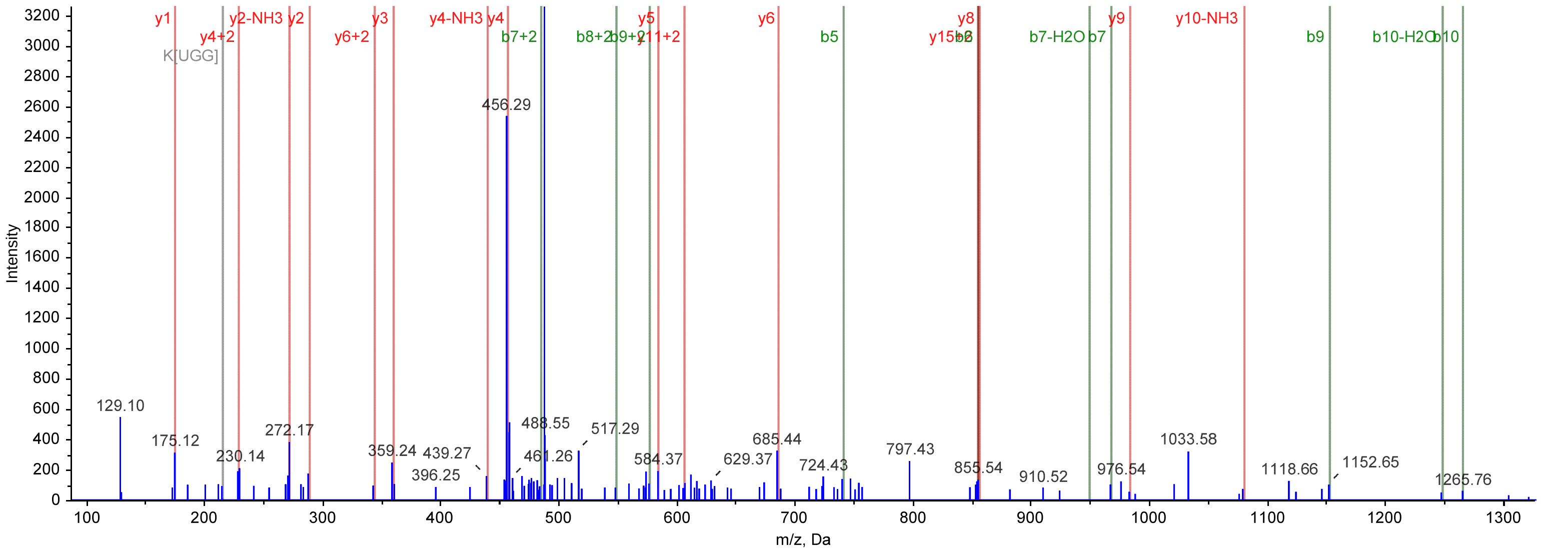

Residue	Immonium	b	b+2	b-H2O	y	y+2	y-NH3
K[UGG]	215.1503	243.1452	122.0762	225.1346	1950.161	975.584	1933.134
I	86.0964	356.2292	178.6183	338.2187	1708.023	854.515	1690.996
L	86.0964	469.3133	235.1603	451.3027	1594.939	797.973	1577.912
R	129.1135	625.4144	313.2108	607.4038	1481.855	741.431	1464.828
D	88.0393	740.4413	370.7243	722.4308	1325.754	663.3804	1308.727
N	87.0553	854.4843	427.7458	836.4737	1210.727	605.867	1193.7
I	86.0964	967.5683	484.2878	949.5578	1096.684	548.8455	1079.657
Q	101.0709	1095.627	548.3171	1077.6164	983.5996	492.3035	966.5731
G	30.0338	1152.648	576.8278	1134.6378	855.5411	428.2742	838.5145
I	86.0964	1265.732	633.3699	1247.7219	798.5196	399.7634	781.4931
T	74.06	1366.78	683.8937	1348.7696	685.4355	343.2214	668.409
K	101.1073	1494.875	747.9412	1476.8645	584.3879	292.6976	567.3613
P	70.0651	1591.928	796.4676	1573.9173	456.2929	228.6501	439.2663
A	44.0495	1662.965	831.9861	1644.9544	359.2401	180.1237	342.2136
I	86.0964	1776.049	888.5282	1758.0385	288.203	144.6051	271.1765
R	129.1135	1932.15	966.5787	1914.1396	175.119	88.0631	158.0924

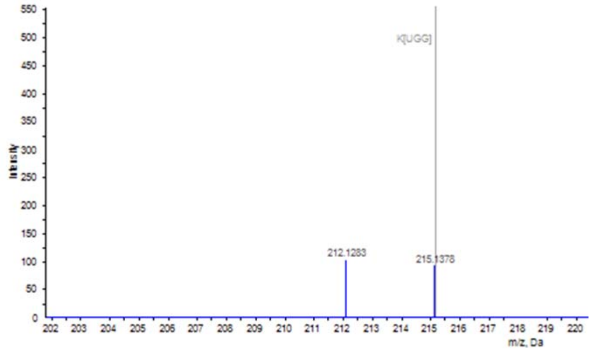

H4:KubTVTSLDVVYALK

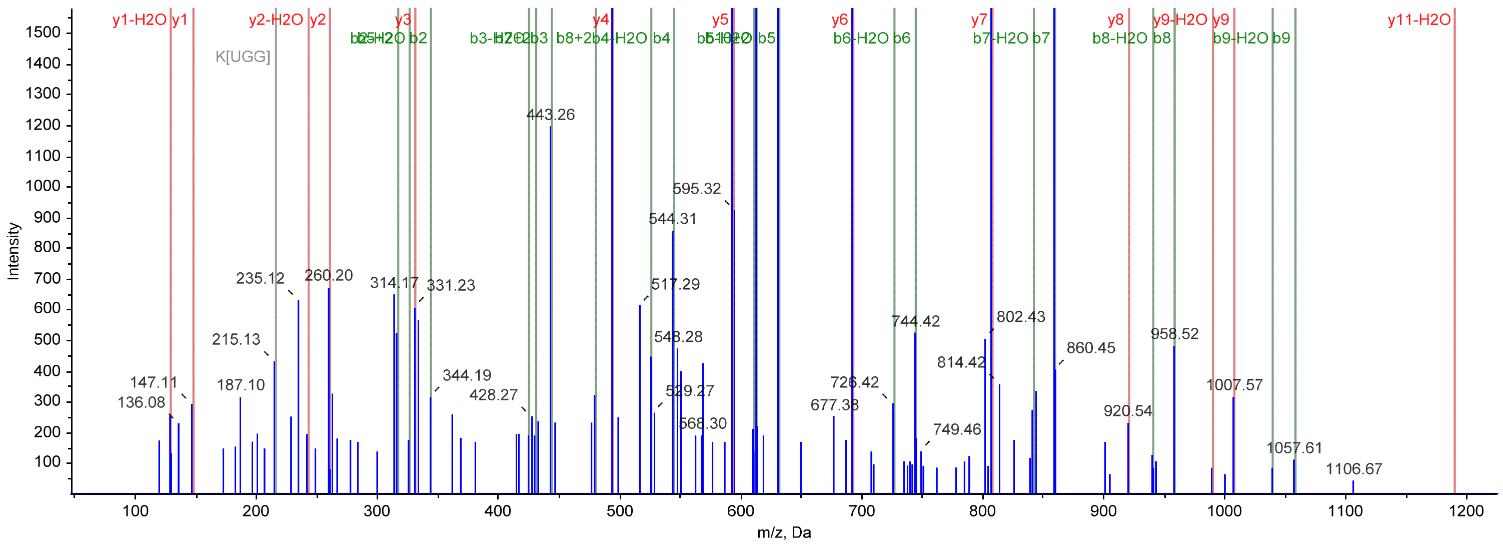

Residue	Immonium	b	b+2	b-H2O	y	y-H2O
K[UGG]	215.1503	243.1452	122.0762	225.1346	1550.8788	1532.868
T	74.06	344.1928	172.6001	326.1823	1308.7409	1290.73
V	72.0808	443.2613	222.1343	425.2507	1207.6933	1189.683
T	74.06	544.3089	272.6581	526.2984	1108.6249	1090.614
S	60.0444	631.341	316.1741	613.3304	1007.5772	989.5666
L	86.0964	744.425	372.7162	726.4145	920.5451	902.5346
D	88.0393	859.452	430.2296	841.4414	807.4611	789.4505
V	72.0808	958.5204	479.7638	940.5098	692.4341	674.4236
V	72.0808	1057.589	529.298	1039.578	593.3657	575.3552
Y	136.0757	1220.652	610.8297	1202.642	494.2973	476.2867
A	44.0495	1291.689	646.3483	1273.679	331.234	313.2234
L	86.0964	1404.773	702.8903	1386.763	260.1969	242.1863
K	101.1073	1532.868	766.9378	1514.858	147.1128	129.1022

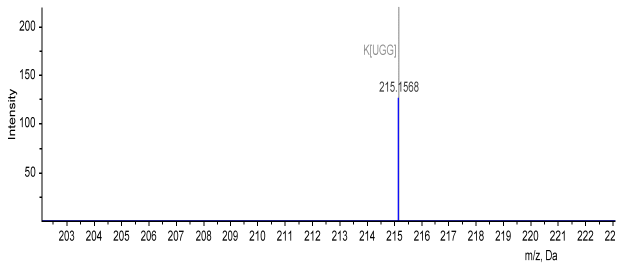

H4:KubTVTSLDVVYALKubR

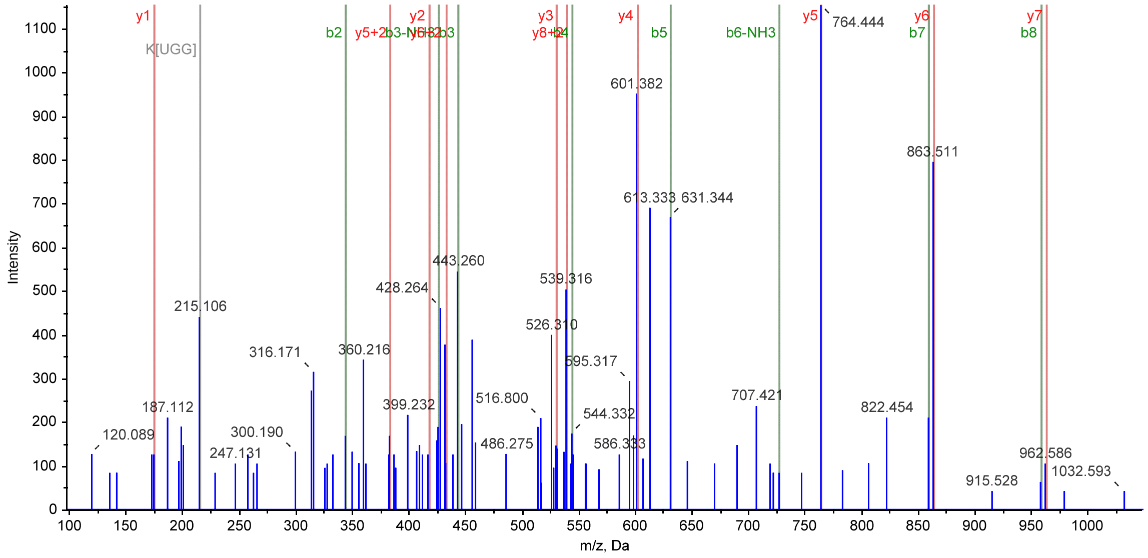

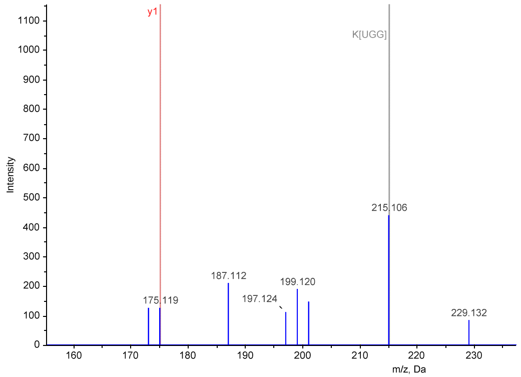

Residue	Immonium	b	b+2	b-H2O	b-NH3	y	y+2	y-H2O	y-NH3
K[UGG]	215.1503	243.1452	122.0762	225.1346	226.1186	1821.023	911.0151	1803.012	1803.996
T	74.06	344.1928	172.6001	326.1823	327.1663	1578.885	789.9461	1560.874	1561.858
V	72.0808	443.2613	222.1343	425.2507	426.2347	1477.837	739.4223	1459.827	1460.811
T	74.06	544.3089	272.6581	526.2984	527.2824	1378.769	689.8881	1360.758	1361.742
S	60.0444	631.341	316.1741	613.3304	614.3144	1277.721	639.3642	1259.711	1260.695
L	86.0964	744.425	372.7162	726.4145	727.3985	1190.689	595.8482	1172.679	1173.663
D	88.0393	859.452	430.2296	841.4414	842.4254	1077.605	539.3062	1059.595	1060.579
V	72.0808	958.5204	479.7638	940.5098	941.4938	962.5782	481.7927	944.5676	945.5516
V	72.0808	1057.589	529.298	1039.578	1040.562	863.5098	432.2585	845.4992	846.4832
Y	136.0757	1220.652	610.8297	1202.642	1203.626	764.4413	382.7243	746.4308	747.4148
A	44.0495	1291.689	646.3483	1273.679	1274.663	601.378	301.1926	583.3675	584.3515
L	86.0964	1404.773	702.8903	1386.763	1387.747	530.3409	265.6741	512.3303	513.3144
K[UGG]	215.1503	1646.911	823.9592	1628.901	1629.885	417.2568	209.1321	399.2463	400.2303
R	129.1135	1803.012	902.0098	1785.002	1785.986	175.119	88.0631	157.1084	158.0924

H4:KTVTSLDVVYALKubR

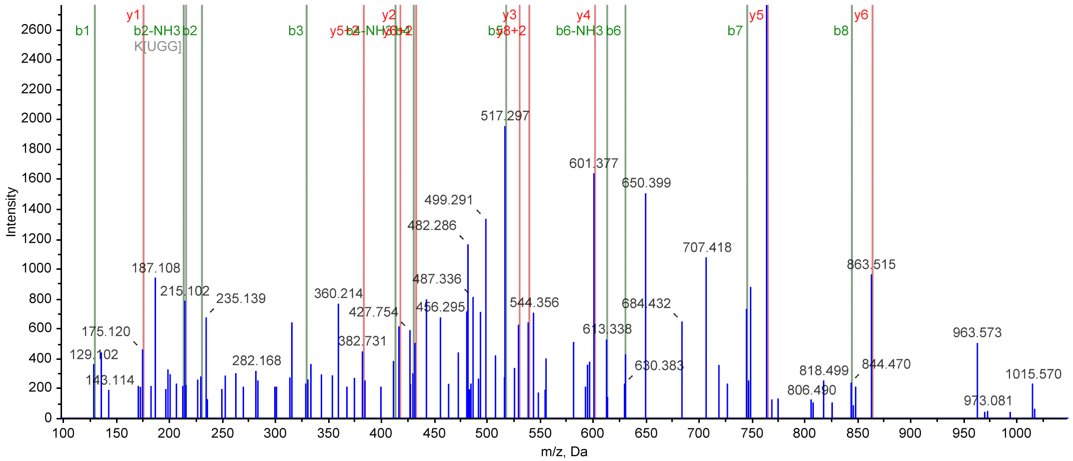

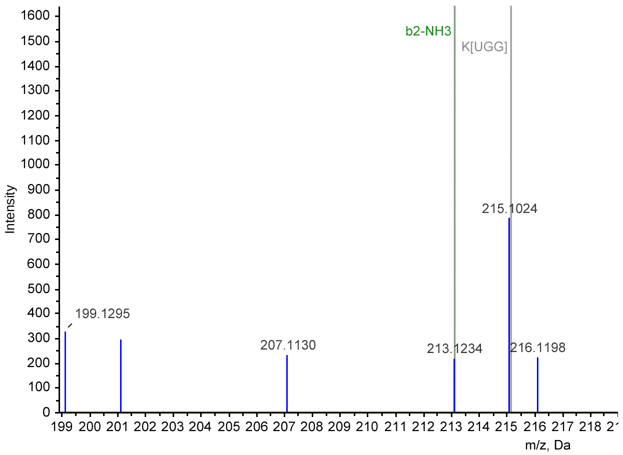

Residue	Immonium	b	b-H2O	b-NH3	y	y+2	y-H2O	y-NH3
K	101.1073	129.1022	111.0917	112.0757	1706.98	853.9936	1688.969	1689.953
T	74.06	230.1499	212.1394	213.1234	1578.885	789.9461	1560.874	1561.858
V	72.0808	329.2183	311.2078	312.1918	1477.837	739.4223	1459.827	1460.811
T	74.06	430.266	412.2554	413.2395	1378.769	689.8881	1360.758	1361.742
S	60.0444	517.298	499.2875	500.2715	1277.721	639.3642	1259.711	1260.695
L	86.0964	630.3821	612.3715	613.3556	1190.689	595.8482	1172.679	1173.663
D	88.0393	745.409	727.3985	728.3825	1077.605	539.3062	1059.595	1060.579
V	72.0808	844.4775	826.4669	827.4509	962.5782	481.7927	944.5676	945.5516
V	72.0808	943.5459	925.5353	926.5193	863.5098	432.2585	845.4992	846.4832
Y	136.0757	1106.609	1088.599	1089.583	764.4413	382.7243	746.4308	747.4148
A	44.0495	1177.646	1159.636	1160.62	601.378	301.1926	583.3675	584.3515
L	86.0964	1290.73	1272.72	1273.704	530.3409	265.6741	512.3303	513.3144
K[UGG]	215.1503	1532.868	1514.858	1515.842	417.2568	209.1321	399.2463	400.2303
R	129.1135	1688.969	1670.959	1671.943	175.119	88.0631	157.1084	158.0924

H4: SubFLESVIR

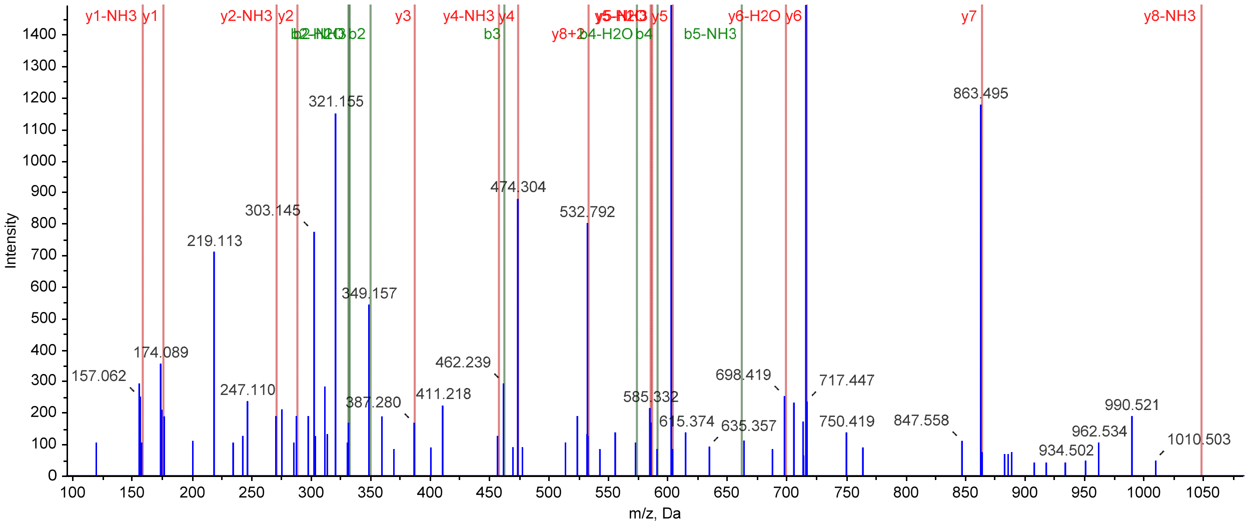

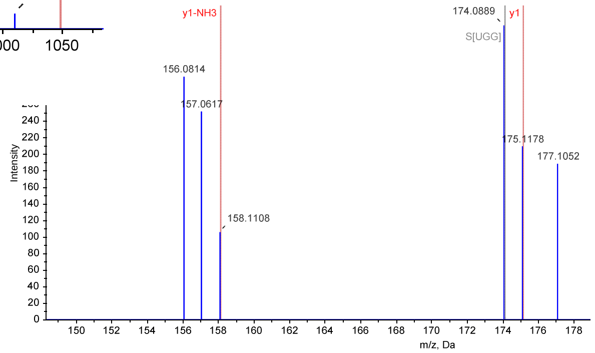

Residue	Immonium	b	b+2	b-H2O	b-NH3	y	y+2	y-H2O	y-[illegible]
S[UGG]	174.0873	202.0822	101.5448	184.0717	185.0557	1064.574	532.7904	1046.563	1047.547
F	120.0808	349.1506	175.079	331.1401	332.1241	863.4985	432.2529	845.488	846.472
L	86.0964	462.2347	231.621	444.2241	445.2082	716.4301	358.7187	698.4196	699.4036
E	102.055	591.2773	296.1423	573.2667	574.2508	603.3461	302.1767	585.3355	586.3195
S	60.0444	678.3093	339.6583	660.2988	661.2828	474.3035	237.6554	456.2929	457.2769
V	72.0808	777.3777	389.1925	759.3672	760.3512	387.2714	194.1394	369.2609	370.2449
I	86.0964	890.4618	445.7345	872.4512	873.4353	288.203	144.6051	270.1925	271.1765
R	129.1135	1046.563	523.7851	1028.552	1029.5364	175.119	88.0631	157.1084	158.0924

H4: TVTSLDVVYALKUbR

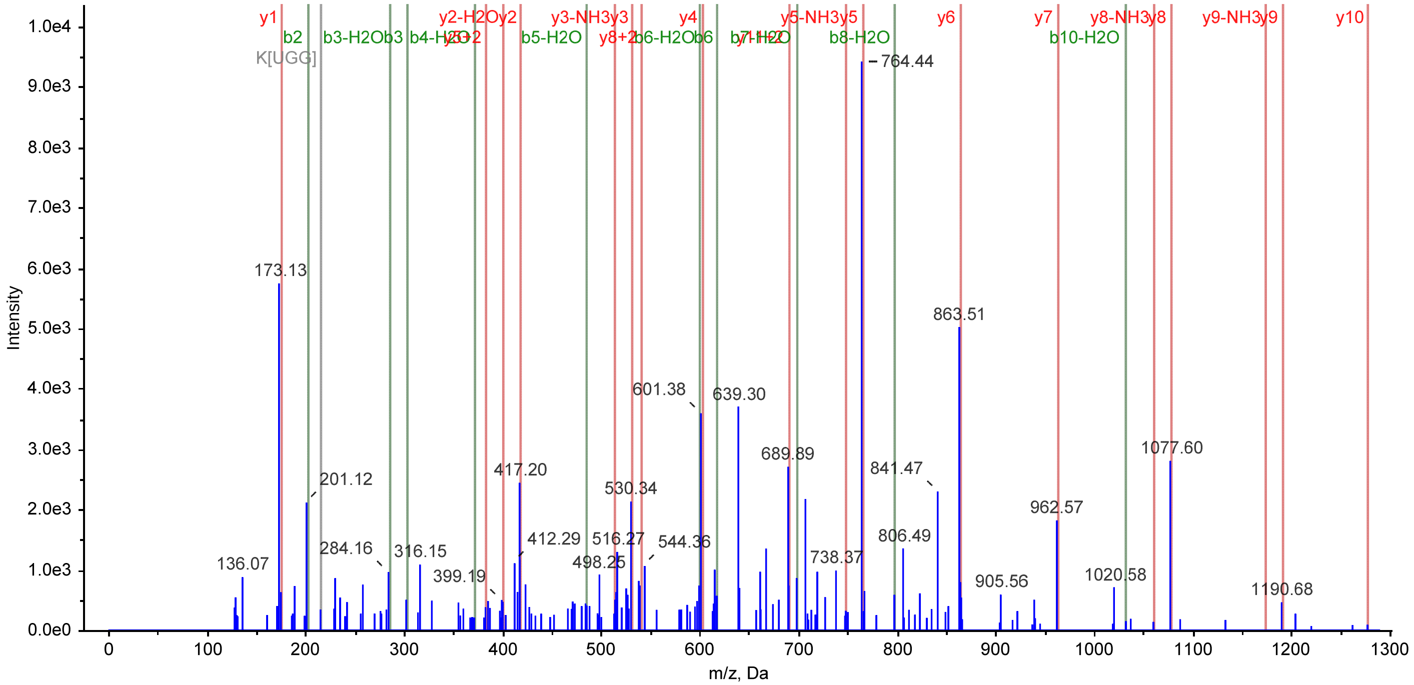

Residue	Immonium	b	b+2	b-H2O	y	y+2	y-H2O	y-NH3
T	74.06	102.055	51.5311	84.0444	1578.885	789.9461	1560.874	1561.858
V	72.0808	201.1234	101.0653	183.1128	1477.837	739.4223	1459.827	1460.811
T	74.06	302.171	151.5892	284.1605	1378.769	689.8881	1360.758	1361.742
S	60.0444	389.2031	195.1052	371.1925	1277.721	639.3642	1259.711	1260.695
L	86.0964	502.2871	251.6472	484.2766	1190.689	595.8482	1172.679	1173.663
D	88.0393	617.3141	309.1607	599.3035	1077.605	539.3062	1059.595	1060.579
V	72.0808	716.3825	358.6949	698.3719	962.5782	481.7927	944.5676	945.5516
V	72.0808	815.4509	408.2291	797.4403	863.5098	432.2585	845.4992	846.4832
Y	136.0757	978.5142	489.7608	960.5037	764.4413	382.7243	746.4308	747.4148
A	44.0495	1049.551	525.2793	1031.541	601.378	301.1926	583.3675	584.3515
L	86.0964	1162.635	581.8213	1144.625	530.3409	265.6741	512.3303	513.3144
K[UGG]	215.1503	1404.773	702.8903	1386.763	417.2568	209.1321	399.2463	400.2303
R	129.1135	1560.874	780.9408	1542.864	175.119	88.0631	157.1084	158.0924

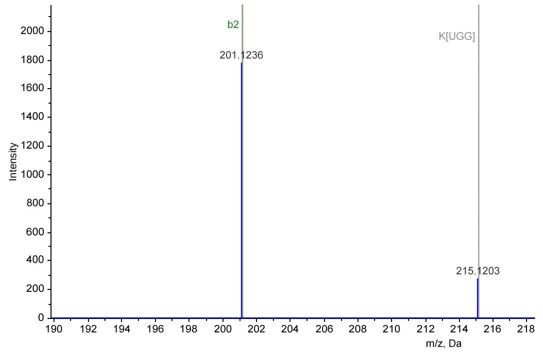

H4: ISGLIYEEVRAVLKubSFLESVIR

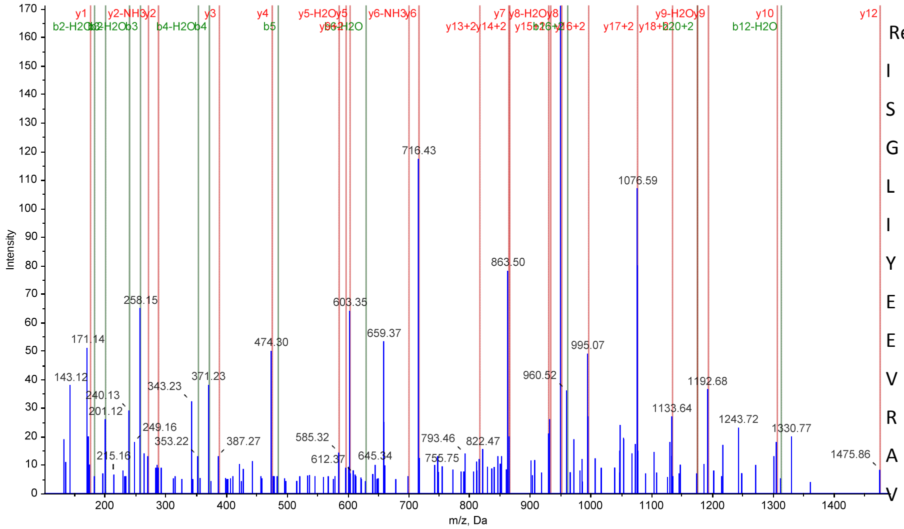

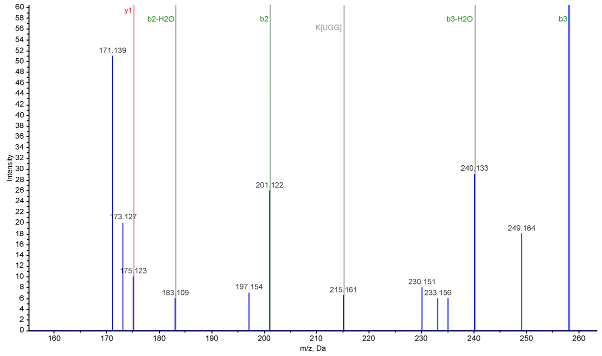

Residue	Immonium	b	b+2	b-H2O	y	y+2	y-H2O	y-NH3
I	86.0964	114.0913	57.5493	96.0808	2635.482	1318.245	2617.471	2618.455
S	60.0444	201.1234	101.0653	183.1128	2522.398	1261.703	2504.387	2505.371
G	30.0338	258.1448	129.5761	240.1343	2435.366	1218.187	2417.355	2418.339
L	86.0964	371.2289	186.1181	353.2183	2378.344	1189.676	2360.334	2361.318
I	86.0964	484.313	242.6601	466.3024	2265.26	1133.134	2247.25	2248.234
Y	136.0757	647.3763	324.1918	629.3657	2152.176	1076.592	2134.166	2135.15
E	102.055	776.4189	388.7131	758.4083	1989.113	995.06	1971.102	1972.086
E	102.055	905.4615	453.2344	887.4509	1860.07	930.5387	1842.06	1843.044
V	72.0808	1004.53	502.7686	986.5193	1731.028	866.0174	1713.017	1714.001
R	129.1135	1160.631	580.8191	1142.62	1631.959	816.4832	1613.949	1614.933
A	44.0495	1231.668	616.3377	1213.658	1475.858	738.4327	1457.848	1458.832
V	72.0808	1330.737	665.8719	1312.726	1404.821	702.9141	1386.81	1387.794
L	86.0964	1443.821	722.4139	1425.81	1305.753	653.3799	1287.742	1288.726
K[UGG]	215.1503	1685.959	843.4829	1667.948	1192.668	596.8379	1174.658	1175.642
S	60.0444	1772.991	886.9989	1754.98	950.5306	475.7689	932.52	933.504
F	120.0808	1920.059	960.5331	1902.048	863.4985	432.2529	845.488	846.472
L	86.0964	2033.143	1017.075	2015.132	716.4301	358.7187	698.4196	699.4036
E	102.055	2162.186	1081.596	2144.175	603.3461	302.1767	585.3355	586.3195
S	60.0444	2249.218	1125.112	2231.207	474.3035	237.6554	456.2929	457.2769
V	72.0808	2348.286	1174.647	2330.276	387.2714	194.1394	369.2609	370.2449
I	86.0964	2461.37	1231.189	2443.36	288.203	144.6051	270.1925	271.1765
R	129.1135	2617.471	1309.239	2599.461	175.119	88.0631	157.1084	158.0924

H2A:LLGNVTIAQGGVLPNIHQNLLPKub

Residue	Immonium	b	b+2	b-H2O	b-NH3	y	y+2	y-H2O	y-NH3
L	86.0964	114.0913	57.5493	96.0808	97.0648	2523.441	1262.224	2505.43	2506.414
L	86.0964	227.1754	114.0913	209.1648	210.1489	2410.357	1205.682	2392.346	2393.33
G	30.0338	284.1969	142.6021	266.1863	267.1703	2297.272	1149.14	2279.262	2280.246
N	87.0553	398.2398	199.6235	380.2292	381.2132	2240.251	1120.629	2222.24	2223.224
V	72.0808	497.3082	249.1577	479.2976	480.2817	2126.208	1063.608	2108.198	2109.182
T	74.06	598.3559	299.6816	580.3453	581.3293	2027.14	1014.074	2009.129	2010.113
I	86.0964	711.44	356.2236	693.4294	694.4134	1926.092	963.5496	1908.081	1909.065
A	44.0495	782.4771	391.7422	764.4665	765.4505	1813.008	907.0076	1794.997	1795.981
Q	101.0709	910.5356	455.7715	892.5251	893.5091	1741.971	871.489	1723.96	1724.944
G	30.0338	967.5571	484.2822	949.5465	950.5306	1613.912	807.4597	1595.902	1596.886
G	30.0338	1024.579	512.7929	1006.568	1007.552	1556.891	778.949	1538.88	1539.864
V	72.0808	1123.647	562.3271	1105.636	1106.62	1499.869	750.4383	1481.859	1482.843
L	86.0964	1236.731	618.8692	1218.721	1219.705	1400.801	700.9041	1382.79	1383.774
P	70.0651	1333.784	667.3955	1315.773	1316.757	1287.717	644.362	1269.706	1270.69
N	87.0553	1447.827	724.417	1429.816	1430.8	1190.664	595.8357	1172.654	1173.638
I	86.0964	1560.911	780.959	1542.9	1543.884	1076.621	538.8142	1058.611	1059.595
H	110.0713	1697.97	849.4885	1679.959	1680.943	963.537	482.2722	945.5265	946.5105
Q	101.0709	1826.028	913.5178	1808.018	1809.002	826.4781	413.7427	808.4676	809.4516
N	87.0553	1940.071	970.5392	1922.061	1923.045	698.4196	349.7134	680.409	681.393
L	86.0964	2053.155	1027.081	2035.145	2036.129	584.3766	292.6919	566.3661	567.3501
L	86.0964	2166.239	1083.623	2148.229	2149.213	471.2926	236.1499	453.282	454.266
P	70.0651	2263.292	1132.15	2245.282	2246.266	358.2085	179.6079	340.1979	341.1819
K[UGG]	215.1503	2505.43	1253.219	2487.419	2488.404	261.1557	131.0815	243.1452	244.1292

H2A:LLGNVTIAQGGVLPNIHQNLLPKub

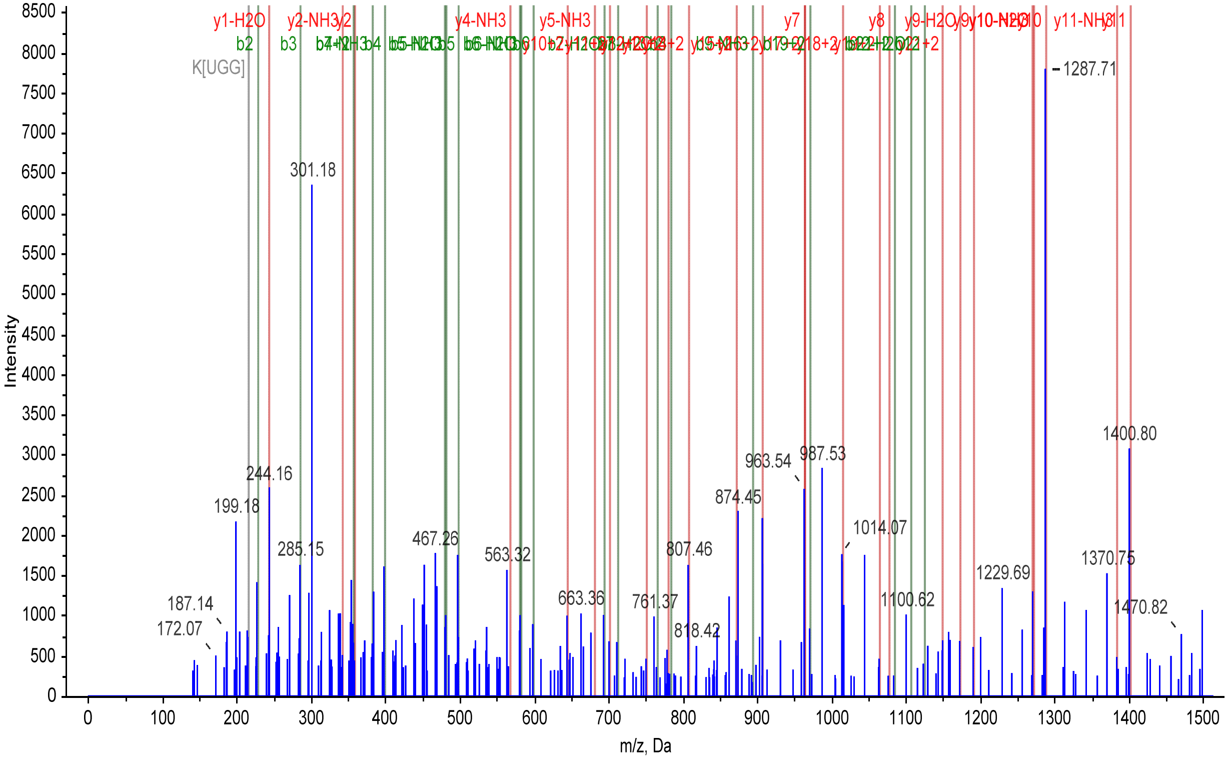

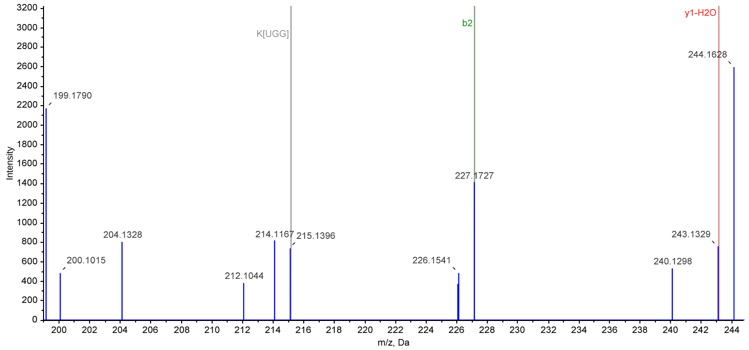

H2A: NDDELNKubLLGNVTIAQGGVLPNIHQNLLPK

Residue	Immonium	b	b+2	b-NH3	y	y+2	y-H2O	y-NH3
N	87.0553	115.0502	58.0287	98.0237	3351.802	1676.405	3333.791	3334.775
D	88.0393	230.0771	115.5422	213.0506	3237.759	1619.383	3219.748	3220.733
D	88.0393	345.1041	173.0557	328.0775	3122.732	1561.87	3104.722	3105.706
E	102.055	474.1467	237.577	457.1201	3007.705	1504.356	2989.695	2990.679
L	86.0964	587.2307	294.119	570.2042	2878.663	1439.835	2860.652	2861.636
N	87.0553	701.2737	351.1405	684.2471	2765.579	1383.293	2747.568	2748.552
K[UGG]	215.1503	943.4116	472.2094	926.385	2651.536	1326.271	2633.525	2634.509
L	86.0964	1056.496	528.7515	1039.469	2409.398	1205.203	2391.387	2392.371
L	86.0964	1169.58	585.2935	1152.553	2296.314	1148.66	2278.303	2279.287
G	30.0338	1226.601	613.8042	1209.575	2183.23	1092.118	2165.219	2166.203
N	87.0553	1340.644	670.8257	1323.618	2126.208	1063.608	2108.198	2109.182
V	72.0808	1439.713	720.3599	1422.686	2012.165	1006.586	1994.155	1995.139
T	74.06	1540.76	770.8837	1523.734	1913.097	957.052	1895.086	1896.07
I	86.0964	1653.844	827.4258	1636.818	1812.049	906.5282	1794.039	1795.023
A	44.0495	1724.881	862.9443	1707.855	1698.965	849.9861	1680.954	1681.938
Q	101.0709	1852.94	926.9736	1835.913	1627.928	814.4676	1609.917	1610.901
G	30.0338	1909.961	955.4843	1892.935	1499.869	750.4383	1481.859	1482.843
G	30.0338	1966.983	983.9951	1949.956	1442.848	721.9275	1424.837	1425.821
V	72.0808	2066.051	1033.529	2049.025	1385.826	693.4168	1367.816	1368.8
L	86.0964	2179.135	1090.071	2162.109	1286.758	643.8826	1268.747	1269.731
P	70.0651	2276.188	1138.598	2259.162	1173.674	587.3406	1155.663	1156.647
N	87.0553	2390.231	1195.619	2373.205	1076.621	538.8142	1058.611	1059.595
I	86.0964	2503.315	1252.161	2486.289	962.5782	481.7927	944.5676	945.5516
H	110.0713	2640.374	1320.691	2623.348	849.4941	425.2507	831.4835	832.4676
Q	101.0709	2768.433	1384.72	2751.406	712.4352	356.7212	694.4246	695.4087
N	87.0553	2882.476	1441.741	2865.449	584.3766	292.6919	566.3661	567.3501
L	86.0964	2995.56	1498.283	2978.533	470.3337	235.6705	452.3231	453.3071
L	86.0964	3108.644	1554.826	3091.617	357.2496	179.1285	339.2391	340.2231
P	70.0651	3205.696	1603.352	3188.67	244.1656	122.5864	226.155	227.139
K	101.1073	3333.791	1667.399	3316.765	147.1128	74.06	129.1022	130.0863

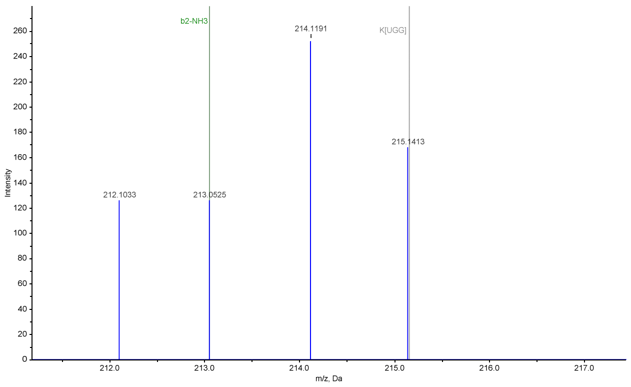

H2A: NDDELNKubLLGNVTIAQGGVLPNIHQNLLPK

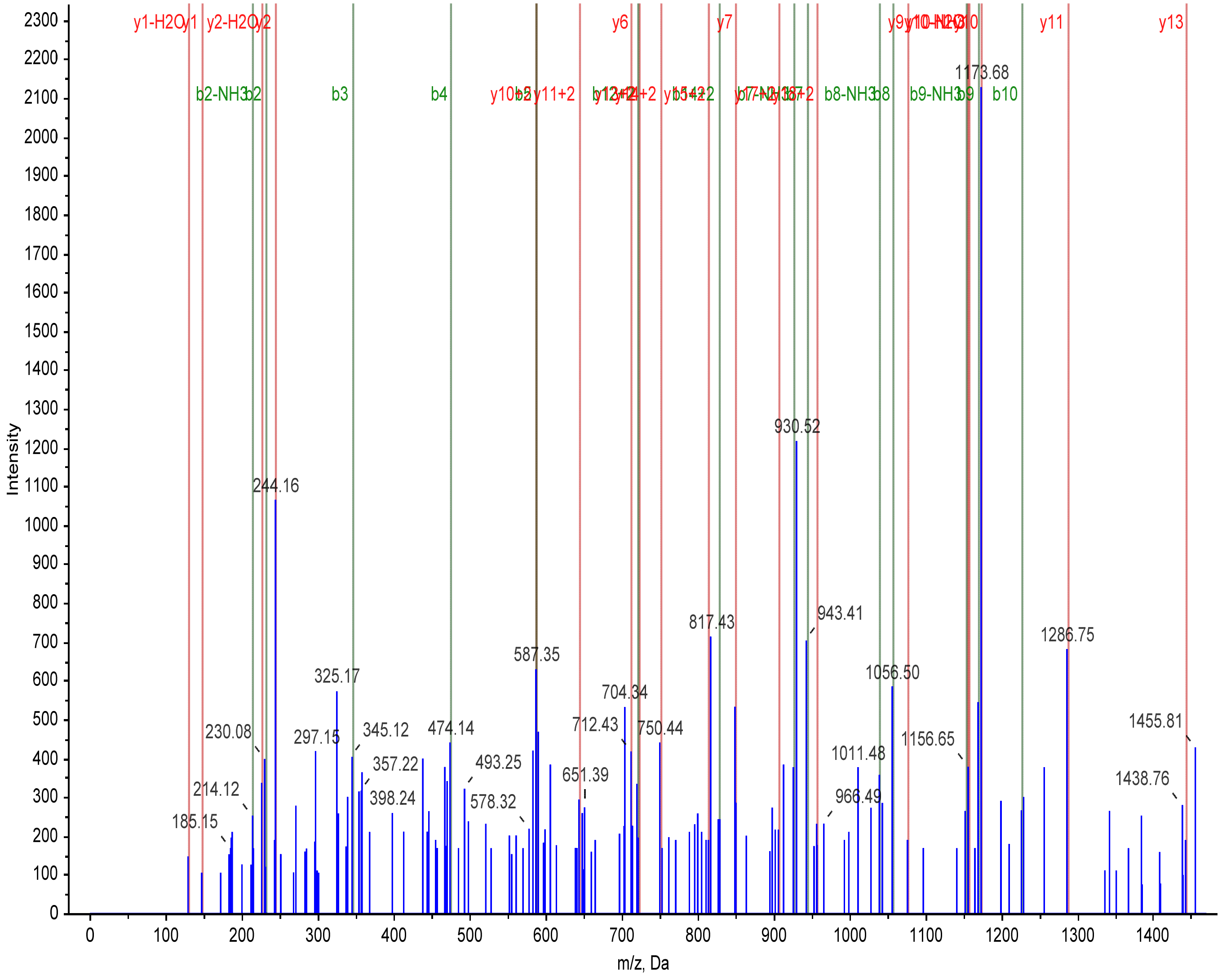

H2A: SAKubAGLTFPVGR

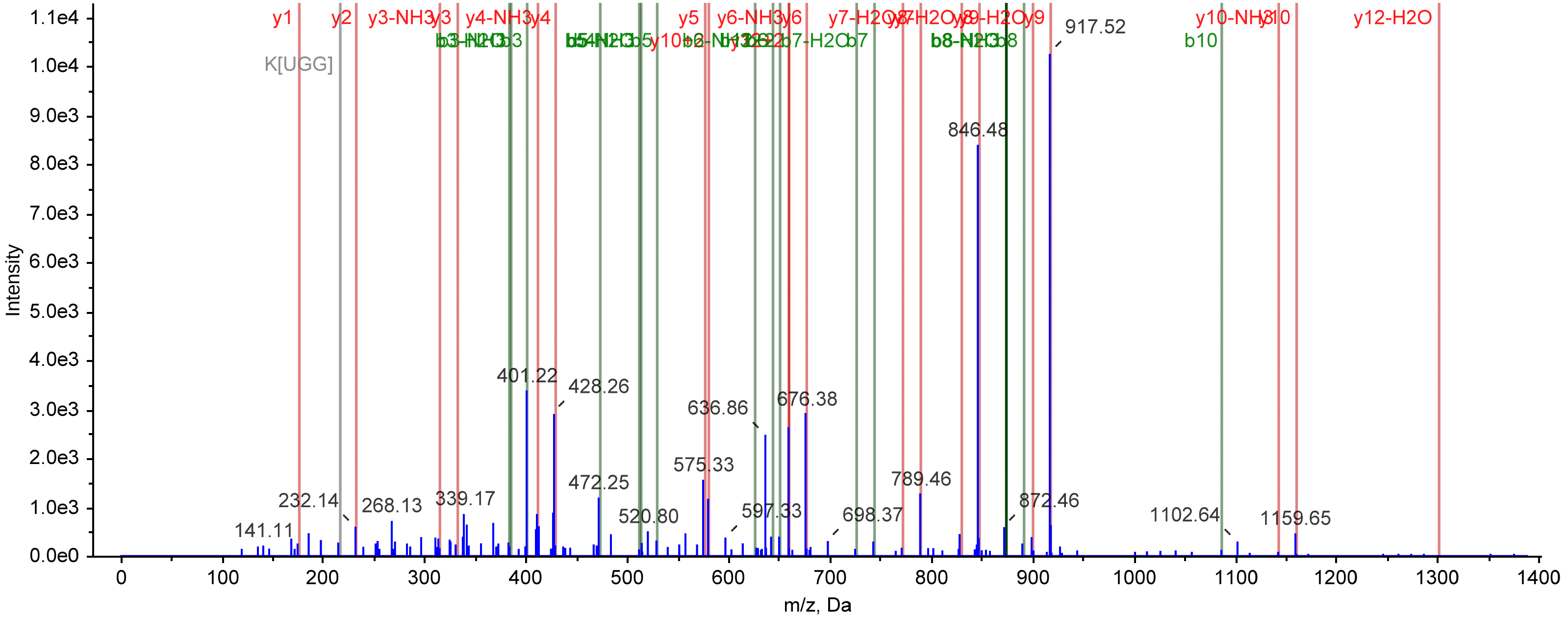

Residue	Immonium	b	b+2	b-H2O	b-NH3	y	y+2	y-H2O	y-NH3
S	60.0444	88.0393	44.5233	70.0287	71.0128	1317.727	659.3673	1299.717	1300.701
A	44.0495	159.0764	80.0418	141.0659	142.0499	1230.695	615.8513	1212.685	1213.669
K[UGG]	215.1503	401.2143	201.1108	383.2037	384.1878	1159.658	580.3327	1141.648	1142.632
A	44.0495	472.2514	236.6293	454.2409	455.2249	917.5203	459.2638	899.5098	900.4938
G	30.0338	529.2729	265.1401	511.2623	512.2463	846.4832	423.7452	828.4726	829.4567
L	86.0964	642.357	321.6821	624.3464	625.3304	789.4618	395.2345	771.4512	772.4352
T	74.06	743.4046	372.206	725.3941	726.3781	676.3777	338.6925	658.3671	659.3511
F	120.0808	890.473	445.7402	872.4625	873.4465	575.33	288.1686	557.3194	558.3035
P	70.0651	987.5258	494.2665	969.5152	970.4993	428.2616	214.6344	410.251	411.235
V	72.0808	1086.594	543.8007	1068.584	1069.568	331.2088	166.1081	313.1983	314.1823
G	30.0338	1143.616	572.3115	1125.605	1126.589	232.1404	116.5738	214.1299	215.1139
R	129.1135	1299.717	650.362	1281.706	1282.69	175.119	88.0631	157.1084	158.0924

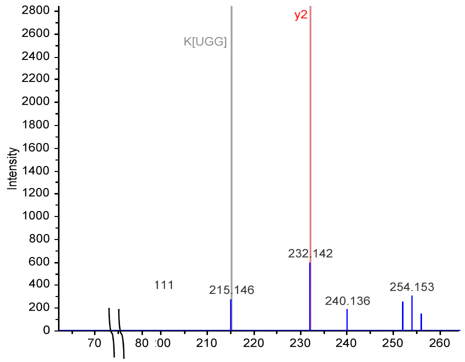

H2B: AVTKubYSSSTQA

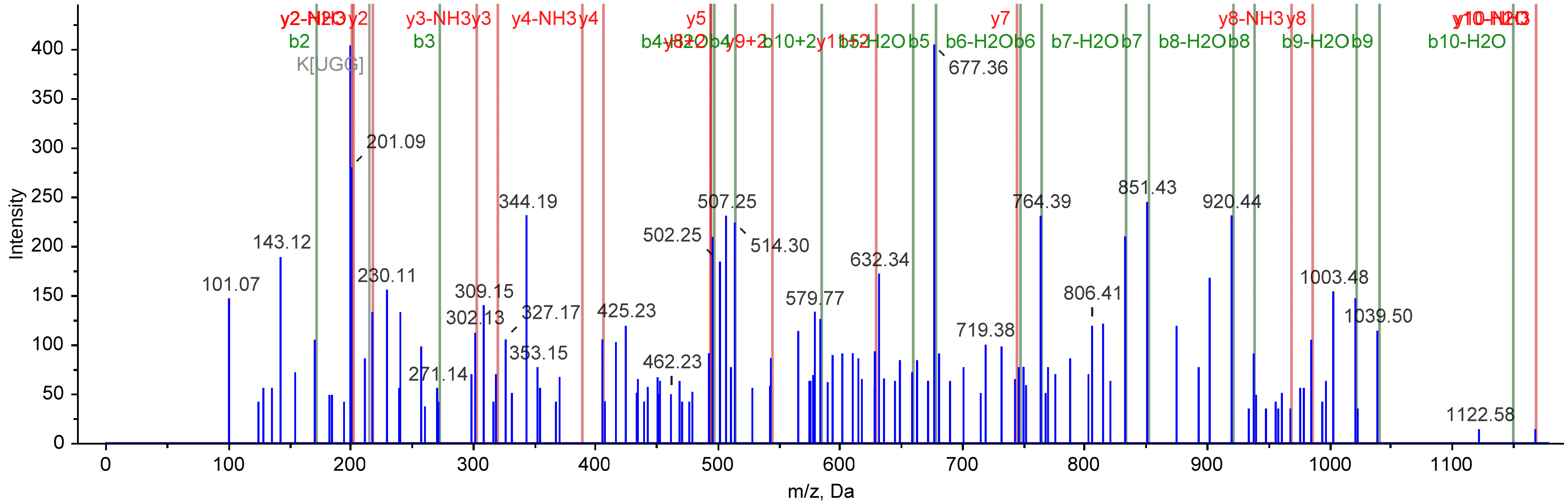

Residue	Immonium	b	b+2	b-H2O	y	y+2	y-H2O	y-NH3
A	44.0495	72.0444	36.5258	54.0338	1256.612	628.8095	1238.601	1239.585
V	72.0808	171.1128	86.06	153.1022	1185.575	593.2909	1167.564	1168.548
T	74.06	272.1605	136.5839	254.1499	1086.506	543.7567	1068.496	1069.48
K[UGG]	215.1503	514.2984	257.6528	496.2878	985.4585	493.2329	967.448	968.432
Y	136.0757	677.3617	339.1845	659.3511	743.3206	372.164	725.3101	726.2941
S	60.0444	764.3937	382.7005	746.3832	580.2573	290.6323	562.2467	563.2307
S	60.0444	851.4258	426.2165	833.4152	493.2253	247.1163	475.2147	476.1987
S	60.0444	938.4578	469.7325	920.4472	406.1932	203.6003	388.1827	389.1667
T	74.06	1039.5055	520.2564	1021.495	319.1612	160.0842	301.1506	302.1347
Q	101.0709	1167.564	584.2857	1149.554	218.1135	109.5604	200.103	201.087
A	44.0495	1238.6012	619.8042	1220.591	90.055	45.5311	72.0444	73.0284

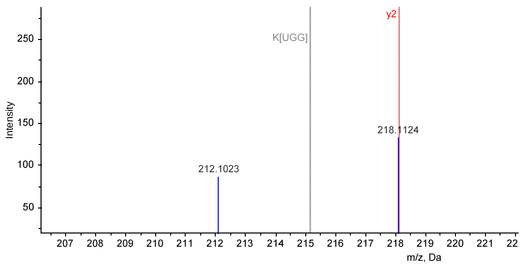

H2B: IATEASKubLAAYNK

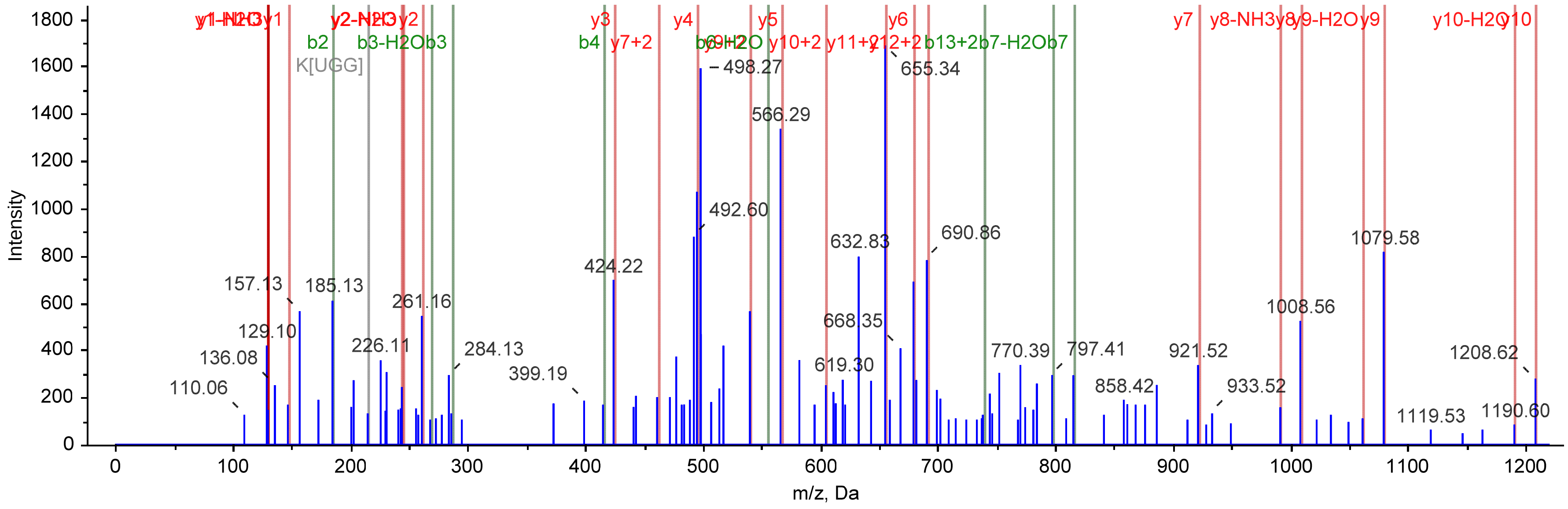

Residue	Immonium	b	b+2	b-H2O	y	y+2	y-H2O	y-NH3
I	86.0964	114.0913	57.5493	96.0808	1493.796	747.4016	1475.785	1476.769
A	44.0495	185.1285	93.0679	167.1179	1380.712	690.8595	1362.701	1363.685
T	74.06	286.1761	143.5917	268.1656	1309.675	655.341	1291.664	1292.648
E	102.055	415.2187	208.113	397.2082	1208.627	604.8171	1190.616	1191.6
A	44.0495	486.2558	243.6316	468.2453	1079.584	540.2958	1061.574	1062.558
S	60.0444	573.2879	287.1476	555.2773	1008.547	504.7773	990.5367	991.5207
K[UGG]	215.1503	815.4258	408.2165	797.4152	921.5152	461.2613	903.5047	904.4887
L	86.0964	928.5098	464.7585	910.4993	679.3774	340.1923	661.3668	662.3508
A	44.0495	999.5469	500.2771	981.5364	566.2933	283.6503	548.2827	549.2667
A	44.0495	1070.5841	535.7957	1052.574	495.2562	248.1317	477.2456	478.2296
Y	136.0757	1233.6474	617.3273	1215.637	424.2191	212.6132	406.2085	407.1925
N	87.0553	1347.6903	674.3488	1329.68	261.1557	131.0815	243.1452	244.1292
K	101.1073	1475.7853	738.3963	1457.775	147.1128	74.06	129.1022	130.0863

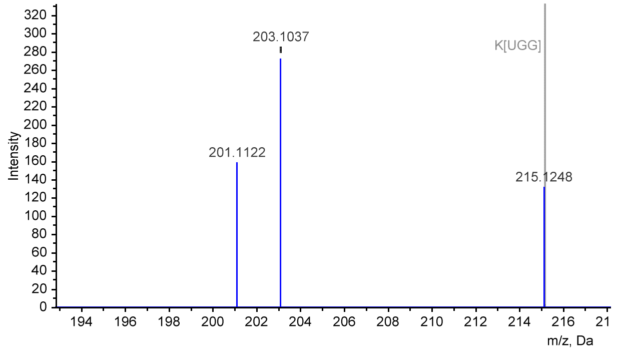

H2B: KubETYSSYIYK

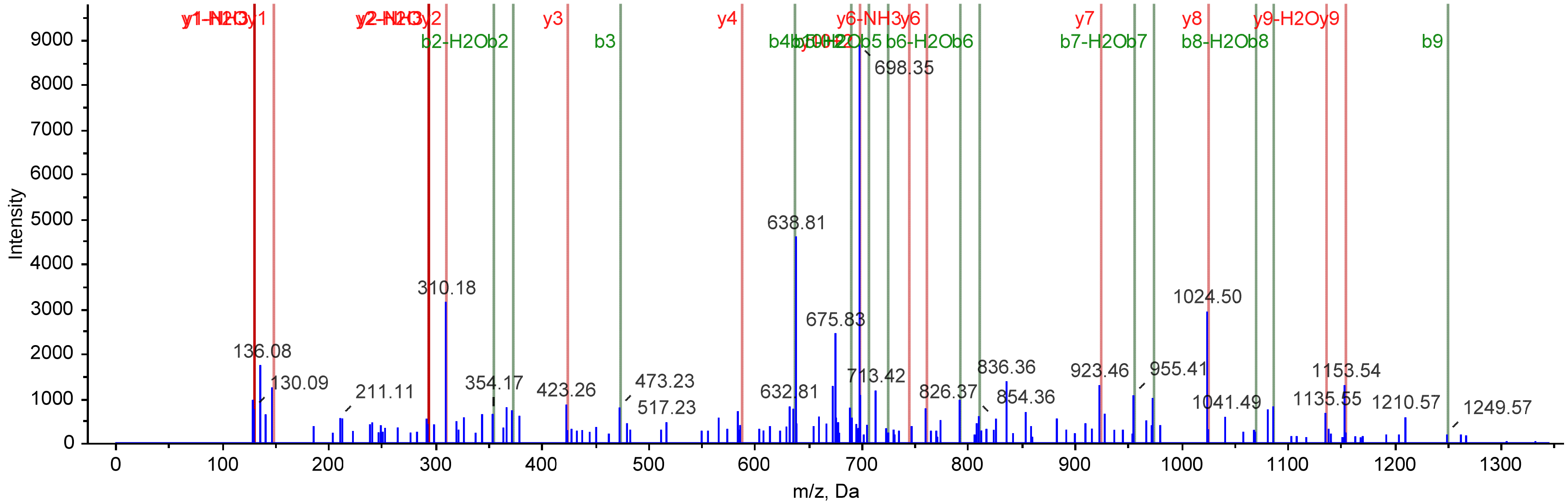

Residue	Immonium	b	b+2	b-H2O	y	y+2	y-H2O	y-NH3
K[UGG]	215.1503	243.1452	122.0762	225.1346	1395.679	698.3432	1377.669	1378.653
E	102.055	372.1878	186.5975	354.1772	1153.541	577.2742	1135.531	1136.515
T	74.06	473.2354	237.1214	455.2249	1024.499	512.7529	1006.488	1007.472
Y	136.0757	636.2988	318.653	618.2882	923.4509	462.2291	905.4403	906.4244
S	60.0444	723.3308	362.169	705.3202	760.3876	380.6974	742.377	743.361
S	60.0444	810.3628	405.6851	792.3523	673.3556	337.1814	655.345	656.329
Y	136.0757	973.4262	487.2167	955.4156	586.3235	293.6654	568.313	569.297
I	86.0964	1086.5102	543.7587	1068.5	423.2602	212.1337	405.2496	406.2336
Y	136.0757	1249.5735	625.2904	1231.563	310.1761	155.5917	292.1656	293.1496
K	101.1073	1377.6685	689.3379	1359.658	147.1128	74.06	129.1022	130.0863

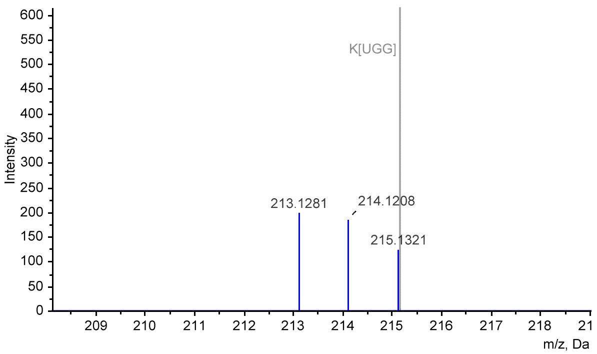

H2B: KETubYSSYIYK

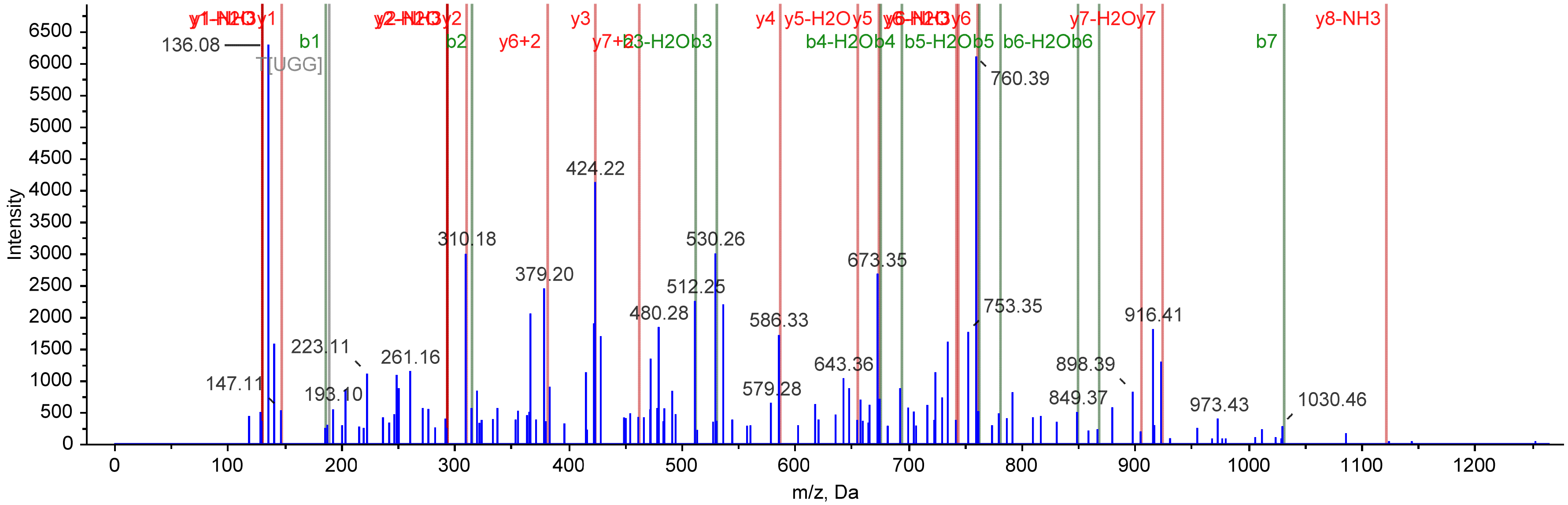

Residue	Immonium	b	b-H2O	y	y+2	y-H2O	y-NH3
K	101.1073	186.1237	168.1131	1452.701	726.8539	1434.69	1435.674
E	102.055	315.1663	297.1557	1267.584	634.2957	1249.574	1250.558
T[UGG]	188.103	530.2569	512.2463	1138.542	569.7744	1120.531	1121.515
Y	136.0757	693.3202	675.3097	923.4509	462.2291	905.4403	906.4244
S	60.0444	780.3523	762.3417	760.3876	380.6974	742.377	743.361
S	60.0444	867.3843	849.3737	673.3556	337.1814	655.345	656.329
Y	136.0757	1030.4476	1012.4371	586.3235	293.6654	568.313	569.297
I	86.0964	1143.5317	1125.5211	423.2602	212.1337	405.2496	406.2336
Y	136.0757	1306.595	1288.5844	310.1761	155.5917	292.1656	293.1496
K	101.1073	1434.69	1416.6794	147.1128	74.06	129.1022	130.0863

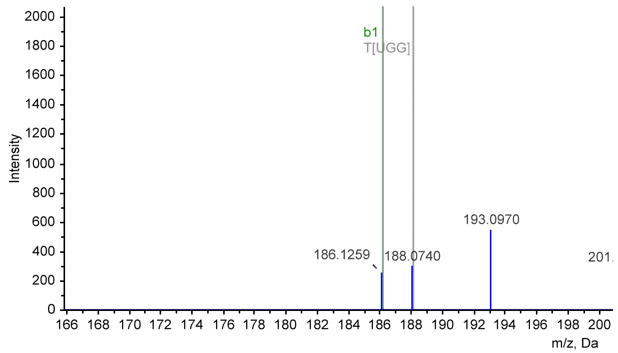

H2B: LILPGELAKubHAVSEGTR

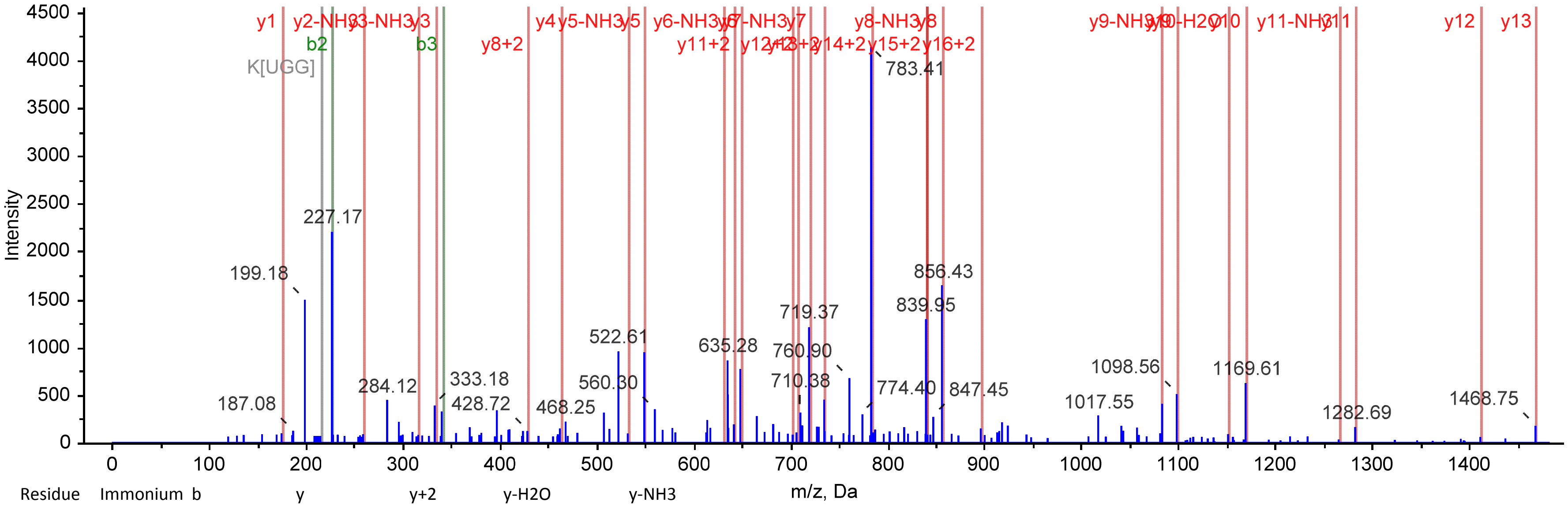

Residue	Immonium	b	y	y+2	y-H2O	y-NH3
L	86.0964	114.0913	1905.0552	953.0313	1887.045	1888.029
I	86.0964	227.1754	1791.9712	896.4892	1773.961	1774.945
L	86.0964	340.2595	1678.8871	839.9472	1660.877	1661.861
P	70.0651	437.3122	1565.803	783.4052	1547.793	1548.777
G	30.0338	494.3337	1468.7503	734.8788	1450.74	1451.724
E	102.055	623.3763	1411.7288	706.368	1393.718	1394.702
L	86.0964	736.4604	1282.6862	641.8468	1264.676	1265.66
A	44.0495	807.4975	1169.6022	585.3047	1151.592	1152.576
K[UGG]	215.1503	1049.6354	1098.565	549.7862	1080.555	1081.539
H	110.0713	1186.6943	856.4272	428.7172	838.4166	839.4006
A	44.0495	1257.7314	719.3682	360.1878	701.3577	702.3417
V	72.0808	1356.7998	648.3311	324.6692	630.3206	631.3046
S	60.0444	1443.8318	549.2627	275.135	531.2522	532.2362
E	102.055	1572.8744	462.2307	231.619	444.2201	445.2041
G	30.0338	1629.8959	333.1881	167.0977	315.1775	316.1615
T	74.06	1730.9436	276.1666	138.587	258.1561	259.1401
R	129.1135	1887.0447	175.119	88.0631	157.1084	158.0924

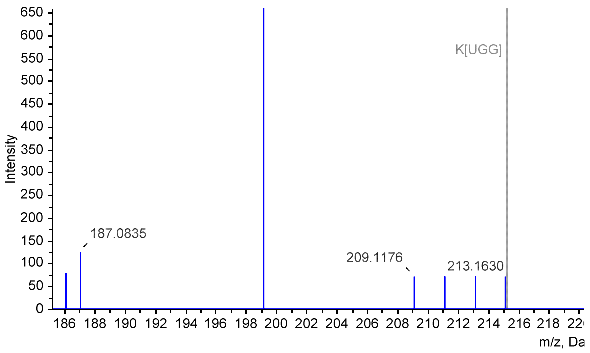

H2B: QTHPDTGISQKub

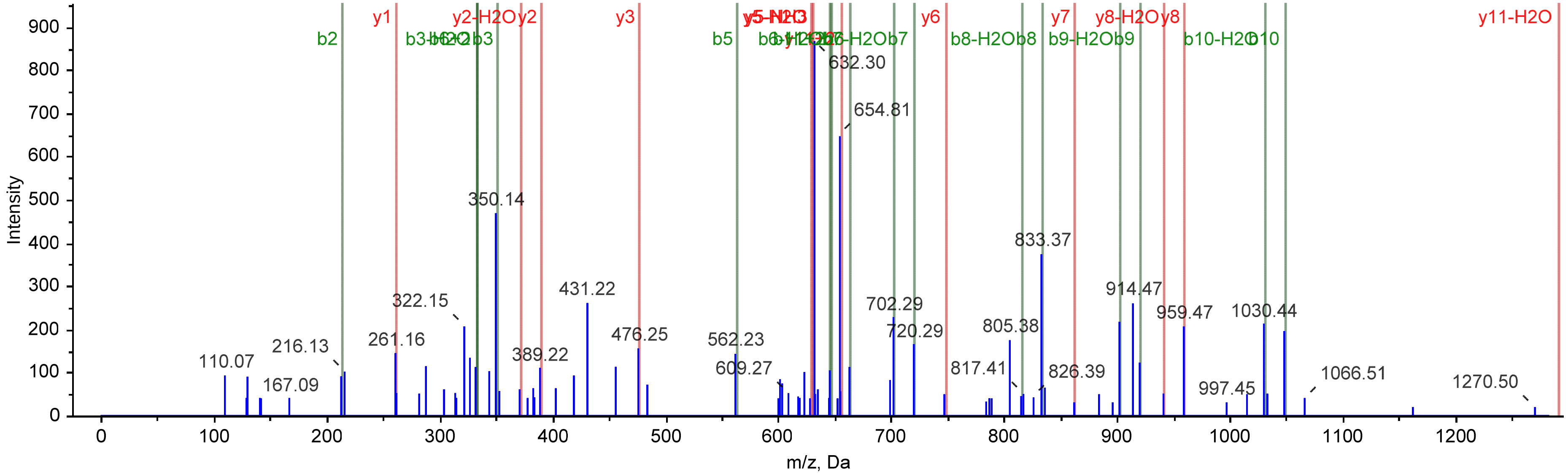

Residue	Immonium	b	b+2	b-H2O	y	y+2	y-H2O	y-NH3
Q	101.0709	112.0393	56.5233	94.0287	1308.618	654.8126	1290.607	1291.591
T	74.06	213.087	107.0471	195.0764	1197.586	599.2966	1179.575	1180.559
H	110.0713	350.1459	175.5766	332.1353	1096.538	548.7727	1078.528	1079.512
P	70.0651	447.1987	224.103	429.1881	959.4793	480.2433	941.4687	942.4527
D	88.0393	562.2256	281.6164	544.215	862.4265	431.7169	844.4159	845.3999
T	74.06	663.2733	332.1403	645.2627	747.3995	374.2034	729.389	730.373
G	30.0338	720.2947	360.651	702.2842	646.3519	323.6796	628.3413	629.3253
I	86.0964	833.3788	417.193	815.3682	589.3304	295.1688	571.3198	572.3039
S	60.0444	920.4108	460.7091	902.4003	476.2463	238.6268	458.2358	459.2198
Q	101.0709	1048.4694	524.7383	1030.459	389.2143	195.1108	371.2037	372.1878
K[UGG]	215.1503	1290.6073	645.8073	1272.597	261.1557	131.0815	243.1452	244.1292

PSH1: SDITTIPALNTTLQQYLSFILEKub

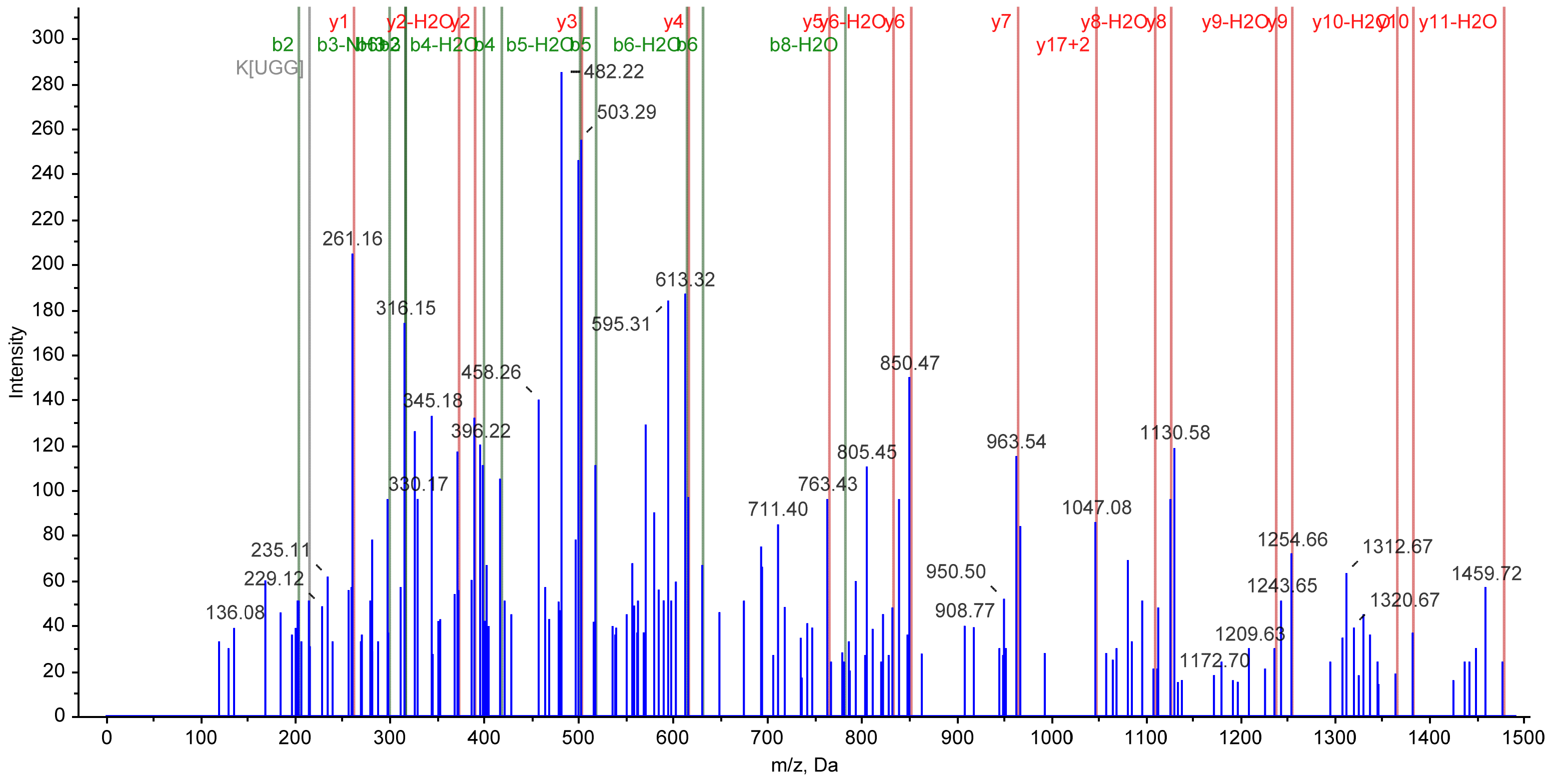

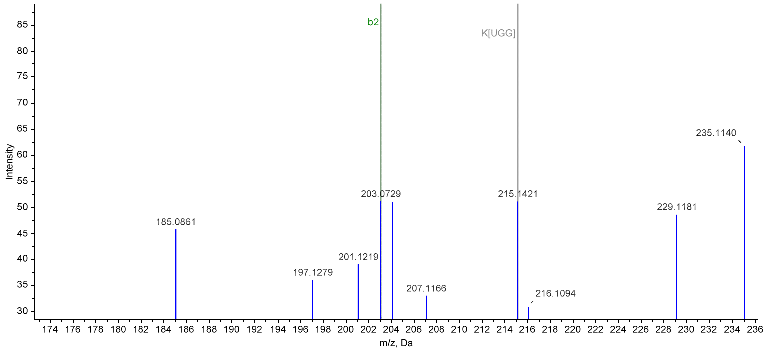

PSH1: SDITTIPALNTTLQQYLSFILEKub

Residue	Immonium	b	b+2	b-H2O	b-NH3	y	y+2	y-H2O
S	60.0444	88.0393	44.5233	70.0287	71.0128	2723.45	1362.229	2705.44
D	88.0393	203.0662	102.0368	185.0557	186.0397	2636.418	1318.713	2618.408
I	86.0964	316.1503	158.5788	298.1397	299.1238	2521.391	1261.199	2503.381
T	74.06	417.198	209.1026	399.1874	400.1714	2408.307	1204.657	2390.297
T	74.06	518.2457	259.6265	500.2351	501.2191	2307.26	1154.133	2289.249
I	86.0964	631.3297	316.1685	613.3192	614.3032	2206.212	1103.61	2188.201
P	70.0651	728.3825	364.6949	710.3719	711.3559	2093.128	1047.068	2075.117
A	44.0495	799.4196	400.2134	781.409	782.3931	1996.075	998.5411	1978.064
L	86.0964	912.5037	456.7555	894.4931	895.4771	1925.038	963.0226	1907.027
N	87.0553	1026.5466	513.7769	1008.536	1009.52	1811.954	906.4805	1793.943
T	74.06	1127.5943	564.3008	1109.584	1110.568	1697.911	849.4591	1679.9
T	74.06	1228.642	614.8246	1210.631	1211.615	1596.863	798.9352	1578.853
L	86.0964	1341.726	671.3666	1323.716	1324.7	1495.816	748.4114	1477.805
Q	101.0709	1469.7846	735.3959	1451.774	1452.758	1382.731	691.8694	1364.721
Q	101.0709	1597.8432	799.4252	1579.833	1580.817	1254.673	627.8401	1236.662
Y	136.0757	1760.9065	880.9569	1742.896	1743.88	1126.614	563.8108	1108.604
L	86.0964	1873.9906	937.4989	1855.98	1856.964	963.551	482.2791	945.5404
S	60.0444	1961.0226	981.0149	1943.012	1943.996	850.4669	425.7371	832.4563
F	120.0808	2108.091	1054.5491	2090.08	2091.065	763.4349	382.2211	745.4243
I	86.0964	2221.1751	1111.0912	2203.165	2204.149	616.3665	308.6869	598.3559
L	86.0964	2334.2591	1167.6332	2316.249	2317.233	503.2824	252.1448	485.2718
E	102.055	2463.3017	1232.1545	2445.291	2446.275	390.1983	195.6028	372.1878
K[UGG]	215.1503	2705.4396	1353.2235	2687.429	2688.413	261.1557	131.0815	243.1452

RT106: KubLDLVFYLSNVDGSPVITLLK

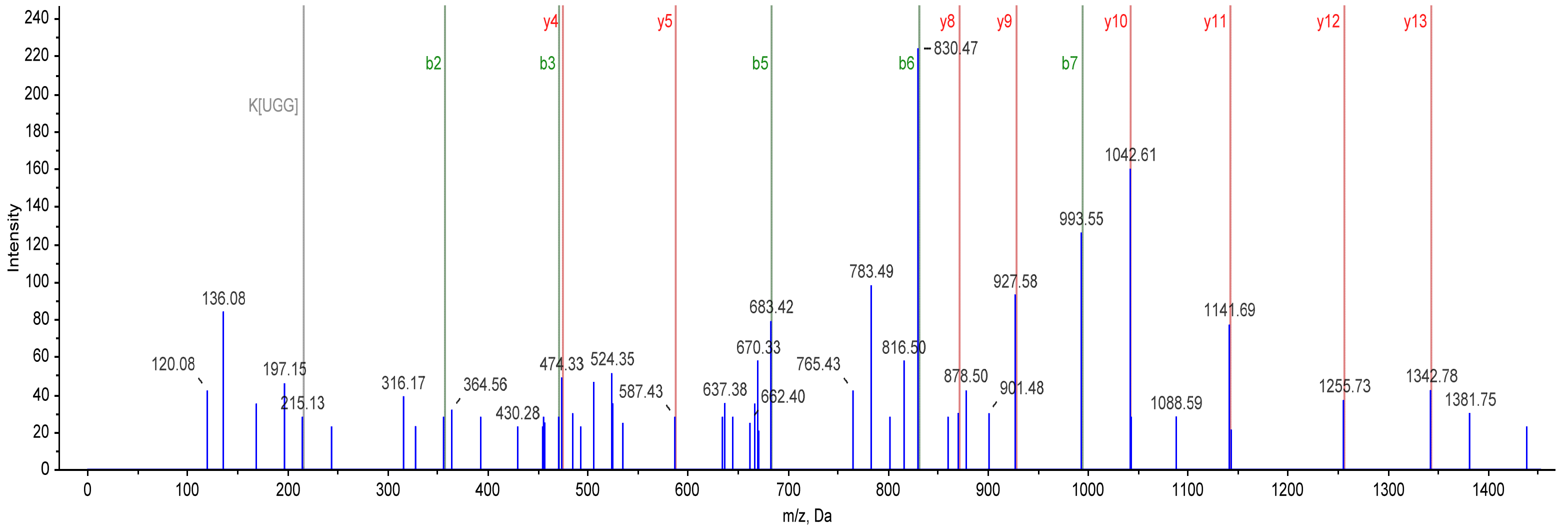

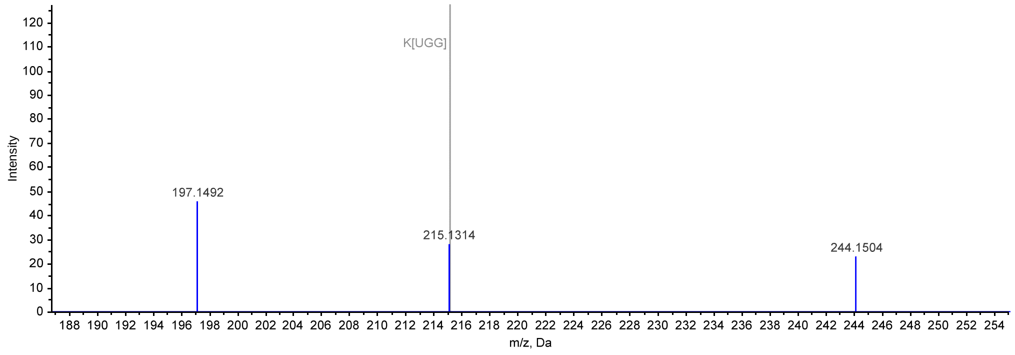

RT106: KubLDLVFYLSNVDGSPVITLLK

Residue	Immonium	b	b-H2O	y
K[UGG]	215.1503	243.1452	225.1346	2448.375
L	86.0964	356.2292	338.2187	2206.237
D	88.0393	471.2562	453.2456	2093.153
L	86.0964	584.3402	566.3297	1978.126
V	72.0808	683.4087	665.3981	1865.042
F	120.0808	830.4771	812.4665	1765.974
Y	136.0757	993.5404	975.5298	1618.905
L	86.0964	1106.6245	1088.6139	1455.842
S	60.0444	1193.6565	1175.6459	1342.758
N	87.0553	1307.6994	1289.6888	1255.726
V	72.0808	1406.7678	1388.7573	1141.683
D	88.0393	1521.7948	1503.7842	1042.614
G	30.0338	1578.8162	1560.8057	927.5873
S	60.0444	1665.8483	1647.8377	870.5659
P	70.0651	1762.901	1744.8905	783.5339
V	72.0808	1861.9694	1843.9589	686.4811
I	86.0964	1975.0535	1957.0429	587.4127
T	74.06	2076.1012	2058.0906	474.3286
L	86.0964	2189.1852	2171.1747	373.2809
L	86.0964	2302.2693	2284.2587	260.1969
K	101.1073	2430.3643	2412.3537	147.1128

RT106: KubQAILTGFK

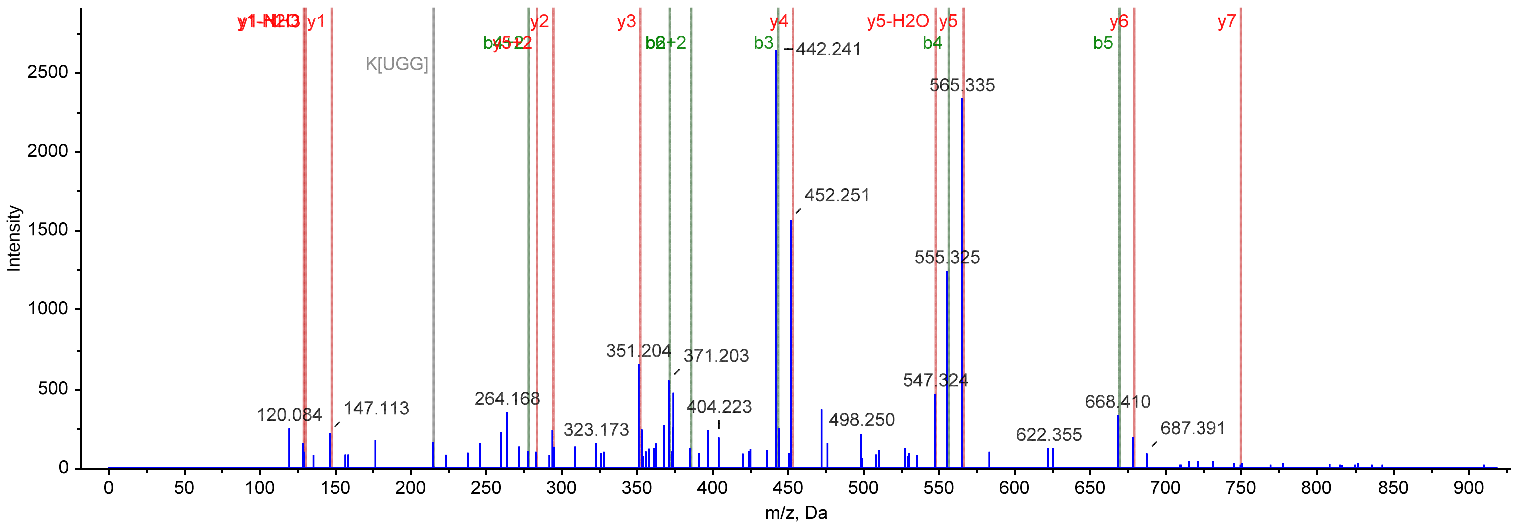

Residue	Immonium	b	b+2	y	y+2	y-H2O	y-NH3
K[UGG]	215.1503	243.1452	122.0762	1119.652	560.3297	1101.642	1102.626
Q	101.0709	371.2037	186.1055	877.5142	439.2607	859.5036	860.4876
A	44.0495	442.2409	221.6241	749.4556	375.2314	731.445	732.4291
I	86.0964	555.3249	278.1661	678.4185	339.7129	660.4079	661.3919
L	86.0964	668.409	334.7081	565.3344	283.1709	547.3239	548.3079
T	74.06	769.4567	385.232	452.2504	226.6288	434.2398	435.2238
G	30.0338	826.4781	413.7427	351.2027	176.105	333.1921	334.1761
F	120.0808	973.5465	487.2769	294.1812	147.5942	276.1707	277.1547
K	101.1073	1101.6415	551.3244	147.1128	74.06	129.1022	130.0863

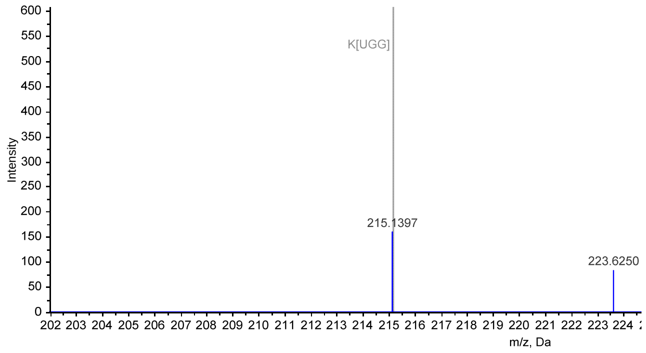

Supplement: Supplementary file 2 — Supplementary material [file mmc2.zip › Supplementary Tables_DiB/Spectrum ubiquitination validation.pdf]
